# Ginsenoside Rb1 induces a pro-neurogenic microglial phenotype via PPARγ activation in male mice exposed to chronic mild stress

**DOI:** 10.1186/s12974-021-02185-0

**Published:** 2021-08-09

**Authors:** Lijuan Zhang, Minmin Tang, Xiaofang Xie, Qiuying Zhao, Nan Hu, Hui He, Gangcai Liu, Shiqi Huang, Cheng Peng, Ying Xiao, Zili You

**Affiliations:** 1 School of Life Science and Technology, Mental Health Center of Chengdu, University of Electronic Science and Technology of China, Chengdu, 610054 China; 2 State Key Laboratory of Southwestern Chinese Medicine Resources, School of Pharmacy, Chengdu University of Traditional Chinese Medicine, Chengdu, 611137 China; 3 School of Medicine, University of Electronic Science and Technology of China, Chengdu, 610054 China

**Keywords:** Ginsenoside Rb1, Major depressive disorder, Microglia, Pro-neurogenic phenotype, Neurogenesis, Antidepressant

## Abstract

**Background:**

Anti-inflammatory approaches are emerging as a new strategy for the treatment of depressive disorders. Ginsenoside Rb1 (GRb1), a major component of Panax ginseng, can inhibit inflammatory cascade and alleviate depressive-like behaviors. Microglia can promote or inhibit adult hippocampal neurogenesis according to their functional phenotypes. Here, we examine whether GRb1 may exert antidepressant effects by promoting a pro-neurogenic phenotype of microglia and thereby increasing neurogenesis.

**Methods:**

The antidepressant effects of GRb1 or the licensed antidepressant imipramine (IMI) were assessed in chronic mild stress (CMS)-exposed male mice. The depressive-like behaviors of mice were evaluated by sucrose preference test, forced swimming test (FST), and tail suspension test (TST). The microglial phenotypes were identified by pro- and anti-inflammatory cytokine expression and morphological properties, analyzed by RT-qPCR, western blotting, and immunofluorescence staining. The effect of GRb1-treated microglia on adult hippocampal neurogenesis in vivo and in vitro was detected using immunofluorescence staining.

**Results:**

Behavioral assessment indicated that GRb1 or IMI treatment alleviated depressive-like behaviors in CMS-exposed mice. Immunofluorescence examination demonstrated that GRb1 induced a pro-neurogenic phenotype of microglia via activating PPARγ in vivo and in vitro, which were effectively reversed by the PPARγ inhibitor GW9662. In addition, GRb1-treated microglia increased the proliferation and differentiation of neural precursor cells.

**Conclusions:**

These findings demonstrated that GRb1 alleviated depressive-like behaviors of CMS-exposed male mice mainly through PPARγ-mediated microglial activation and improvement of adult hippocampus neurogenesis.

**Supplementary Information:**

The online version contains supplementary material available at 10.1186/s12974-021-02185-0.

## Background

Major depressive disorder (MDD), a major public health burden, remains underdiagnosed and undertreated [1]. As a disease involving neuroplasticity dysfunction, MDD is related to chronic neuroinflammation impairment [2]. The imbalance of the inflammatory process is observed in rodent models of stress-induced depression, with the increase of pro-inflammatory cytokines interleukin (IL)-1β, IL-6, and IL-18 and the decrease of anti-inflammatory cytokines IL-10, transforming growth factor (TGF)-β, and IL-4 [3, 4].

Microglia play a key role in immune surveillance of the central nervous system (CNS). Microglia polarization is divided into the classical M1-like (pro-inflammatory) phenotype or the alternative M2-like (anti-inflammatory) phenotype. Microglial M1-like status produces pro-inflammatory mediators, such as inducible nitric oxide synthase, IL-1β, and TNF-α [5]. In contrast, the microglia M2-like phenotype enhances expression of anti-inflammatory cytokines, such as IL-4, TGF-β, and IL-13 [6]. Activated microglia exert different effects on the proliferation and differentiation of neural precursor cells (NPCs) in vivo and in vitro. The pro-inflammatory phenotype impairs the survival and proliferation of NPCs, whereas the anti-inflammatory phenotype increases the generation of new neurons [7].

There is a close relationship between depression pathogenesis and adult hippocampal neurogenesis impairment. The hippocampus is involved in mood regulation and changes in neurogenic activity which is associated with MDD [8]. In MDD patients, hippocampus atrophy has been reported [9]. In animal models of depression, the depressive-like behaviors are associated with the impairment of adult hippocampal neurogenesis [10]. Therefore, modulating the switch of microglial phenotypes from neurotoxicity to pro-neurogenesis should be a strategy for treating depression. It has been reported that anti-inflammatory agents such as minocycline inhibited the microglial pro-inflammatory phenotype and promoted neurogenesis [11]. And activation of microglia by anti-inflammatory cytokines IL4 enhanced neurogenesis and ameliorated depressive-like behaviors [12].

Currently, pharmaceutical treatment is able to reduce the symptoms of depression to some extent. However, varying degrees of toxic side effects limit its long-term clinical application in the treatment of MDD. Traditional Chinese medicines are widely used in China and other countries for the treatment of mental disorders [13]. The herbal medicine salvianolic acid B enhanced neurogenesis and ameliorated depressive-like behaviors [14]. Ginseng has been widely applied to treat various conditions in China and other countries for over 5000 years, with beneficial effects on immune functions and stress resilience [15]. It is shown high safety of ginseng in clinical prescription [16]. Clinical observations show that ginseng plays a significant antidepressant effect [17].

Ginsenoside is one of the most biologically active ingredients in ginseng, with a triterpenoid glycoside structure. Depending on their aglycone moieties, these glycosides are divided into either the 20(S)-protopanaxadiol group (PPD) or 20(S)-protopanaxatriol [18]. Ginsenoside Rb1 (GRb1) is a typical PPD-type saponin. GRb1, one of the main components of Ginseng, has various neuropharmacological effects, including modulating monoamine neurotransmitters, reconstructing neuronal plasticity, regulating the function of the hypothalamic-pituitary-adrenal axis, and anti-inflammatory activities [19, 20]. GRb1 exerts significant antidepressant effects in chronic mild stress (CMS)-exposed rodents [21]. Already known that GRb1 regulates activation of microglia, protecting neurons from inflammatory, oxidative injury and promoting neurogenesis [22, 23]. Few studies have focused on the relationship between antidepressant effects of GRb1 and microglia-mediated neuroinflammatory processes. It is plausible that shifts of microglial phenotype with GRb1 could enhance adult hippocampal neurogenesis.

The peroxisome proliferator-activated receptors (PPARs) are divided into three subtypes, including PPARα, PPARβ/δ, and PPARγ [24]. PPARs are involved in the regulation of inflammatory conditions. Specifically, PPARγ has a crucial role in regulating CNS immune responses and injury repair [25]. Microglia polarize into anti-inflammatory M2-like phenotype through the PPARγ pathway [26]. The shifts of M1-like microglia to M2-like phenotype with PPARγ agonists downregulate pro-inflammatory mediators and upregulate pro-neurogenic factors in stress-exposed animals [27]. Conversely, the PPARγ antagonist, GW9662, inhibits the polarization of microglia to M2-like phenotype [28]. Here, we tested the hypothesis that GRb1 alleviated depressive-like behaviors mainly through PPARγ-mediated transition in microglial phenotype and enhancing adult hippocampus neurogenesis in CMS-exposed mice.

## Methods

### Mice/animals

Adult male C57BL/6J mice 8 weeks old (weighing 18–22 g) were obtained from the Laboratory Animal Center of the Sichuan Academy of Medical Sciences (Chengdu, China). The mice were housed individually under controlled conditions (temperature 23 ± 1.5°C, humidity 65 ± 5%, specific pathogen free, single cage) on a 12-h light/dark cycle (7 PM to 7 AM). Mice were acclimated in 27.5 × 15.5 × 18.5 cm plastic cages with sterile cotton wood sawdust. All experimental procedures were approved by the Ethics Committee of the University of Electronic Science and Technology of China and carried out in strict accordance with the National Institutes of Health Guide for the Care and Use of Laboratory Animals (8th edition, revised 2010).

### Chronic mild stress

Male mice were subjected to CMS as described previously [29]. Briefly, mice were subjected to 1 or 2 kinds of random stressors per day. The stressors included cage tilting (45°, 24 h), reversal of the light-dark cycle (24 h), food or water deprivation (12 h), empty or wet cage (12 h), lights-off (3 h), restraint (2 h), cage shaking (1 h), tail clamping (15 min), and ice water stimulation (5 min).

### Drug administration

GRb1 (purity ≥ 97%, C54H92O23, Cat# P0088) was purchased from the Pureone Biotechnology Company of Shanghai. Lipopolysaccharide (LPS, *E. coli*, 0127: B8) were purchased from Sigma-Aldrich Chemical Co. (St Louis, MO, USA) and soluble in phosphate-buffered saline (PBS, Servicebio, Cat# G4202). The experiment administered the following treatments once per day for 4 weeks (at 16:00 h): Saline, GRb1 (20 mg/kg/d, given as a 2 mg/ml solution in 0.9% saline, intragastric administration; Herbpurify, Chengdu, China), or imipramine hydrochloride (IMI, 20 mg/kg/d, intraperitoneally (i.p.); Sigma-Aldrich, Darmstadt, Germany). A subset of stressed animals was pretreated with PPARγ inhibitor GW9662 (1 mg/kg/day in 1% DMSO, i.p., 28 d; Med Chem Express, Monmouth Junction, NJ, USA), then treated 1 h later with GRb1 (control mice were administrated with 1% DMSO solution in 0.9% saline). The doses of GRb1 and IMI were chosen based on previous studies [30, 31]. After intragastric administration for 0.5 h, 1 h, 2 h, 3 h, 12 h, 24 h, and 48 h, the concentration of GRb1 in hippocampus tissues was detected by LC-MS/MS technique.

### Behavioral tests

#### Locomotor activity test

The locomotor activity of mice was evaluated using a mouse autonomic activity tester (Techman Software-zz6, Chengdu, China) in a quiet environment. In each trial, each mouse was placed in a chamber to acclimatization for 5 min. The numbers of movements and standing were recorded during a 10-min test period. After each trial, the apparatus was cleaned using 75% ethyl alcohol.

#### Sucrose preference test and body weight measurement

The SPT was performed as described previously [32]. Before the test, mice were habituated to consume 1% sucrose solution for 24 h. In the test, mice were deprived of food and water for 12 h, then provided with two containers of 1% sucrose and the same amount of water for 2 h. Sugar preference (%) = sugar consumption (g)/[sugar consumption (g) + water consumption (g)] × 100%. The SPT and body weight was performed weekly.

#### Tail suspension test

Each mouse was placed on the end of a rod suspended 30 cm above a tabletop and placed in an individual compartment. The mice were continuously monitored for 6 min using a computer-assisted video camera system (TST-100 Tail suspension Analysis System, Techman Soft, Chengdu, China). The duration of immobility during 6 min was analyzed.

#### Forced swim test

The FST was carried out as described previously [33]. Briefly, the glass cylinders (height 21 cm, diameter 12 cm, volume 1000 ml) were filled with tap water (25 ± 2 °C). Each mouse was monitored for 6 min using a computer-assisted video camera system (FST-100 Forced Swimming Analysis System, Techman Soft, Chengdu, China). Duration of immobility during the last 4 min was analyzed. FST was performed after all other behavioral assays.

### Cell cultures

#### Primary microglial culture

Primary cultures of microglia from neonatal C57BL/6J mice brain (P0–P3 neonates) were prepared as described previously [34]. Briefly, neonates were decapitated under sterile conditions, the scalp and skull cut off, and the brain tissue removed. And brain tissue was placed in a dish containing cold D-Hank’s solution, pH 7.2, without calcium or magnesium (Gibco, Cat# C14175500BT). The entire brain region was dissociated into a single-cell suspension using 0.25% pancreatin (Gibco, Cat# 25200056). The mixed glial cells were cultured for 1 week in DMEM/F12 (Gibco, Cat# C11330500BT) containing 10% fetal bovine serum (Gibco). The isolated microglia were activated with PBS (Servicebio, Cat# G4202) or LPS (100 μg/ml; Sigma-Aldrich) in the presence or absence of (10, 20, 40) μg/ml GRb1. Some cultures were pretreated for 1 h with 10 μM GW9662, then treated with GRb1 for 24 h, and finally with LPS for 24 h. The collected microglia were transferred to a 6-well plate (2 × 10^5^ cells/cm^2^) for subsequent analysis.

#### NPC culture

NPCs from C57BL/6J mice (P0–P3, *n* = 30) were isolated and cultured. Disinfection of infant mice was following the procedure described above in “Primary microglial culture,” and subsequently, the hippocampus tissue was dissociated from sagittal brains in ice-cold DMEM/F12, then digested using 0.25% pancreatin. The digested cells were divided into two parts. One part of the cells (5 × 10^4^ cells/cm^2^) was incubated in a proliferation medium [high-glucose DMEM/F12, 20 ng/ml epidermal growth factor (Peprotech, Cat# 500-P174G), 20 ng/ml fibroblast growth factor (Peprotech, Cat# 450-33), 40 ng/ml N2 (Gibco, Cat# 17502-048), and 80 ng/ml B27 supplement (Gibco, Cat# 17504-04)]. The second (5 × 10^4^ cells/cm^2^) was incubated in a differentiation medium (high-glucose DMEM/F12, 40 ng/ml N2, 80 ng/ml B27 supplement, and 10% fetal bovine serum). To detect the proliferation of NPCs in vitro, cultured cells were incubated with 10 μM bromodeoxyuridine (BrdU, 2 h, 37 °C, i.p.; Sigma-Aldrich, Cat# B5002) before NPCs were fixed.

#### Conditioned microglial medium for NPC culture

Primary microglia were first treated for 24 h with 20 μg/ml GRb1, then with PBS or 100 μg/ml LPS for 24 h. Some cultures were pretreated for 1 h with 10 μM GW9662, then treated for 24 h with GRb1, and finally treated for 24 h with LPS. These media from microglia were harvested and used as the conditioned medium to treat primary NPC cultures. Therefore, there were five NPC culture conditions: PBS-conditioned microglial medium (CM), GRb1-conditioned microglial medium (GRb1-CM), LPS-conditioned microglial medium (LPS-CM), LPS + GRb1-conditioned microglial medium (LPS+GRb1-CM), and LPS + GRb1 + GW9662-conditioned microglial medium (LPS+GRb1+GW-CM). NPC proliferation was analyzed after 24 h in the conditioned medium, while NPC differentiation was analyzed after 10 days in the conditioned medium. Areas with the highest cell density were imaged with × 20 on a Zeiss confocal microscope (LSM 800, Germany). Data were analyzed by an investigator blinded to animal treatment.

#### RNA extraction and reverse transcription-quantitative PCR

In accordance with the previous method [35], brain tissues were sufficiently perfused to exclude the interference of peripheral blood on experimental results. Briefly, mice were anesthetized with pentobarbital diluted in 0.9% saline (50 mg/kg, i.p.; R&D Systems). Brain tissues were collected after intracardial perfusion with 120 ml cold 0.9% saline solution. Both the hippocampus and cortex were dissected out. Each hemisphere was collected separately for subsequent analyses. Brain tissues were homogenized by the GeneUP Total RNA Mini Kit (Biotechrabbit, Cat# BR0702303). Total RNA (10 ng) was converted to cDNA by reverse transcription with TaKaRa reagent (Takara, Cat# 6210A). The following primer pairs were used: β-actin, 5′-CCG TGA AAA GAT GAC CCA GAT C-3′ and 5′-CAC AGC CTG GAT GGC TAC GT-3′; IL-1β, 5′-CCA GCA GGT TAT CAT CAT CAT CC-3′ and 5′-CTC GCA GCA GCA CAT CAA C-3′; tumor necrosis factor (TNF)-α, 5′-TAC TGA ACT TCG GGG TGA TTG GTC C-3′ and 5′-CAG CCT TGT CCC TTG AAG AGA ACC-3′; TGF-β, 5′-GAC CGC AAC AAC GCC ATC TA-3′ and 5′-GGC GTA TCA GTG GGG GTC AG-3′; Arginase (Arg)-1, 5′-AGA CAG CAG AGG AGG TGA AGA G-3′ and 5′-CGA AGC AAG CCA AGG TTA AAG C-3′; PPARγ, 5′-CCC TGG CAA AGC ATT TGT AT-3′ and 5′-CAC CTC TTT GCT CTG CTC CT-3′.

The reaction mixture for RT-qPCR consisted of 1 μl template cDNA, 0.3 μl primer, and 5 μl SsoFast EvaGreen Supermix (Bio-Rad, California, USA) in a total volume of 10 μl. Data were reported as a fold increase in mRNA levels relative to β-actin.

#### Western blotting

Protein extraction, tissue processing, and western blot analysis were based on the described method [36]. The protein extraction kit was as follows: Total Protein Extraction kit (Sangon Biotech, Cat# 786-225), Nucleoprotein Extraction Kit (Sangon Biotech, no. c500009), Protein concentration of extracts BCA kit (Beyotime Institute of Biotechnology). Equal amounts of protein (2 μg/μl, 30 μg) were separated by 12% SDS/PAGE gel electrophoresis, then transferred to nitrocellulose membranes. The membranes were covered with 5% skim milk and incubated for 2 h. Then, membranes with the target protein were incubated overnight at 4 °C with rabbit antibodies against PPARγ (1:2,000; Abcam, 52 KDa, Cat# ab59256), rabbit antibodies against activated PPARγ (p-PPARγ) (Ser112, 1:500; Thermo Fisher Scientific, 54 KDa, Cat# PA5-36763), or GAPDH (1:1,000; 37 KDa, Cat# JM-3777-100). Lamin B (1:1,000; Wanleibio, 67 KDa, Cat# WL01775). The secondary antibodies goat anti-rabbit IgG (1:10,000; Thermo Fisher Scientific, Cat# 31460) were incubated for 2 h. Primary and secondary antibodies were all diluted with 5% skimmed milk. Densitometry of protein bands was performed using ImageJ (version 1.45J; National Institutes of Health, Bethesda, MD, USA).

#### Immunofluorescence

To assay the proliferation of NPCs in the brain, mice received intraperitoneal injections of BrdU (50 mg/kg, i.p., dissolved in 0.9% saline at a concentration of 5 mg/ml) once every 12 h for a total of 6 injections at the 8th week of the experiment and sacrificed after the last injection. To examine the differentiation of NPCs, animals were injected with BrdU (50 mg/kg, i.p.) once every 12 h for a total of 6 injections at the 1th week of our experiment, and sacrificed after 8 weeks. Mice were anesthetized with 1% pentobarbital sodium and perfused transcardially with 120 ml of 0.9% saline followed by 120 ml of 4% paraformaldehyde. Dissected brains were fixed in 4% paraformaldehyde for 48 h, then dehydrated in 10%, 20%, 30% sucrose solution at 4 °C for 24 h each. For immunostaining against DCX, Iba1, and PPARγ, p-PPARγ. nuclei were stained with 4′,6-diamidin-2-phenylindol (DAPI, Roche). The primary antibodies included rat anti-BrdU (1:500; Abcam, Cat# ab 6326), goat anti-DCX (1:400; Santa Cruz, Cat# sc-271390), The secondary antibodies (1:1,000; Jackson ImmunoResearch) included AffiniPure Donkey Anti-Goat IgG, (Cat# 705-585-003), AffiniPure Donkey Anti-Rabbit IgG (Cat# 711-545-152). AffiniPure Donkey Anti-Rat IgG, (Cat# 705-585-003).

### Morphometry of microglia

Images of hippocampal or cerebral cortical slices (1024 × 1024 pixels) were obtained using a Zeiss confocal microscope and the Plan-Apochromat × 63/1.40 NA oil-immersion DIC M27 objective (Zeiss). The mouse brains were sliced into 35-μm-thick slices and chosen to sample every sixth section based on unbiased counting methods [37]. The brain slice per well was selected sequentially in a 1:6 ratio for a mouse, with each brain slice including intact hippocampus and cortex. Microglial density was determined by dividing the number of cells by total volume (mm^3^). Microglial morphology was quantified using skeleton analysis method [38]. Images of each slice were obtained using a Zeiss confocal microscope. The resulting images were skeletonized using the ImageJ software. Image analyses of relative fluorescence intensity were used to quantify the relative expression of PPARγ by the ImageJ software, and the value was normalized to that of the Ctrl group.

### The quantification of neurogenesis regions

The number of BrdU^+^ cells and DCX^+^ cells in the DG was quantified on the basis of the Cavalieri principle with every sixth section of the hippocampus at × 20 objective [39]. In each section, the number of cells was divided by per mm^2^ DG in that section. To estimate the total volume of subregions in the hippocampus, hippocampal slices were chosen according to Cavalieri’s method, and every sixth section was stained with a DAPI antibody at a × 40 magnification. We performed the volume measurements of DG and granular cell layer (GCL) in the hippocampal area of 12 sections per animal using the ImageJ software. The volume of subregions was obtained by multiplying the sum of section areas per animal by 35 μm.

### Statistical analyses

Experiments were performed with at least three samples and three times independently. Samples and animals were randomly allocated to experimental groups. The data and statistical analysis complied with the documentation requirements. All experiments were performed in a blinded manner. All data were expressed as mean ± SEM. Statistical analyses were performed using SPSS for Windows ® (version 17; Chicago, USA). In sucrose preference and body weight from the week 4 to 8 group, the Ctrl and CMS+GRb1 groups at the 4th week and 8th week were assessed by three-way repeated measures (ANOVA). CMS and CMS+IMI groups at the 4th week and 8th week were analyzed by two-way repeated measures (ANOVA). The CMS+GRb1 and CMS+GRb1+GW groups at the 4th week and 8th week were performed using two-way repeated measures (ANOVA). A post hoc test was applied only when the F value reached significance and there was no significance in the homogeneity of variance. Differences among three or more groups were assessed using one-way or two-way analysis of variance as appropriate, followed by the Bonferroni test as post hoc. The level of confidence was set at 95% (*P* < 0.05). To exclude the misinterpretation of results by the difference in the area of the region of interest, the number of proliferating cells in mice, or the total number of cells in each image, normalization of the data was carried out for some analyses in the study.

## Results

### GRb1 alleviates depressive-like behaviors in male mice exposed to CMS

**Fig. 1:**
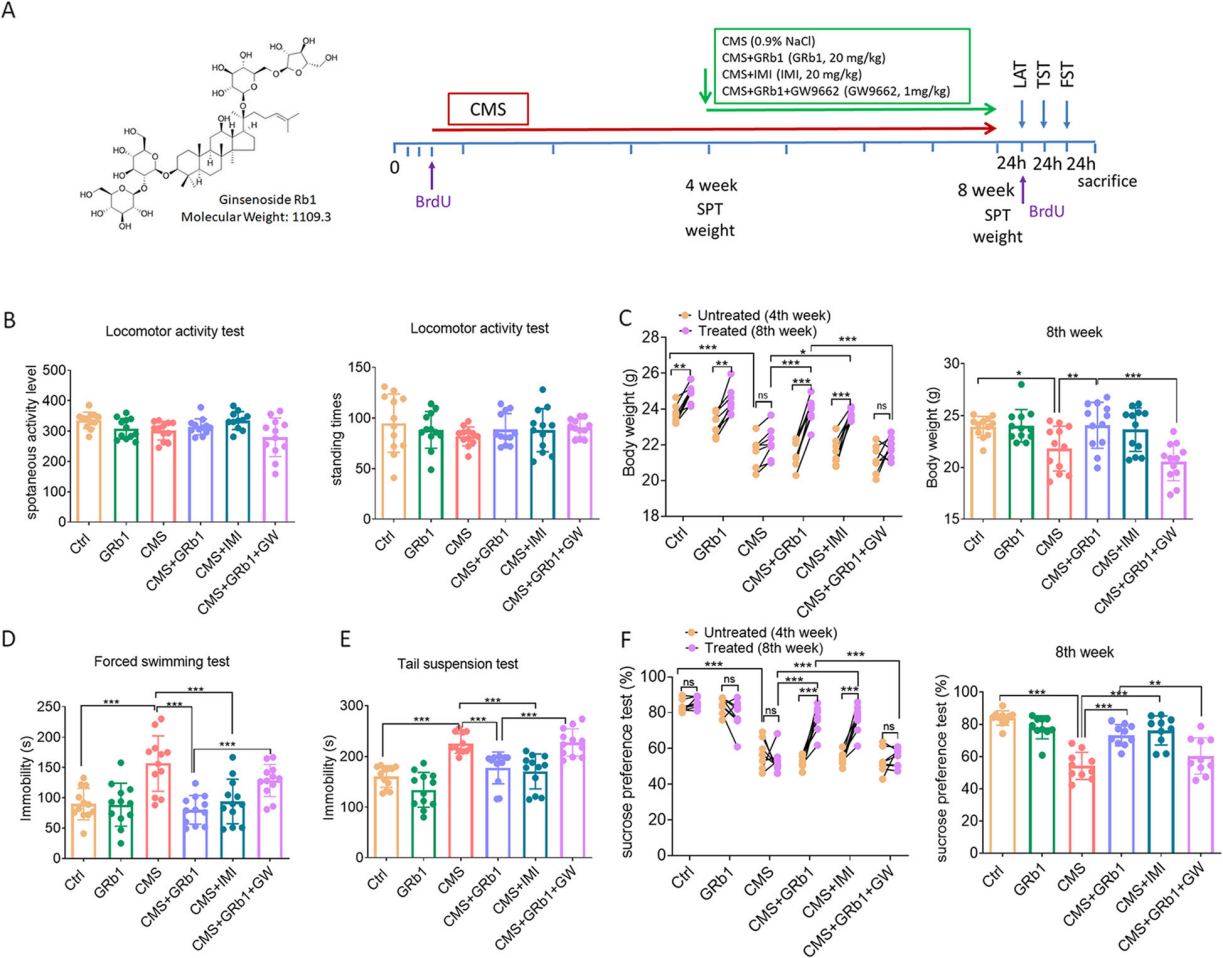
GRb1 alleviates depressive-like behaviors in male mice exposed to CMS. (**A**) Ginsenoside Rb1 chemical structure and experimental flow chart (**B**) Locomotor activity at week 8. (**C**) Comparison of body weight between week 4 and 8, and comparison of weight at week 8 among these groups. (**D**, **E**) Duration of immobility in forced swimming and tail suspension tests at week 8. (**F**) Comparison of sucrose preference between week 4 and 8, and comparison of preference at week 8 among these groups (*n* = 12 mice/group). The statistical results are shown in Table S2. *P < 0.05, ** *P* < 0.01, *** *P* < 0.001

Previous pharmacokinetics and pharmacodynamics experiments have shown that GRb1 plays a role in a variety of CNS disorders [40]. Our experiment showed that GRb1 could be detected in the hippocampus by the LC-MS/MS technique (Fig. S1, Table S1), suggesting that GRb1 was available in the CNS. In the present study, we adopted the CMS paradigm, one of the most extensively validated and realistic models of depression. Following exposure to CMS for 8 weeks, C57BL/6J mice were treated with GRb1 at the 4th week (Fig. 1A). We first assessed the locomotor ability of mice. In spontaneous activity, there was no statistical difference in activity level or standing time among groups, indicating that the drug treatment had no significant effect on motor ability (Fig. 1B). The weight gain test and sucrose preference test are performed individually to exclude the effect of individual differences on the results of the experiment. The weight gain was inhibited by CMS exposure at the 4th and 8th week. The CMS-induced reduction of weight gain was attenuated by GRb1 and IMI at week 8 (Fig. 1C). The FST and TST were performed to test the desperate behaviors of mice. Chronic stress caused mice to remain immobile for longer periods, whereas the GRb1-treated and IMI-treated mice decreased immobility time in both TST and FST (Fig. 1D, E). The sucrose preference test was commonly used to evaluate anhedonia in animals. The CMS groups showed a reduction in sucrose consumption between weeks 4 and 8. Both GRb1- and IMI-treated mice displayed an increase in sucrose consumption compared with the CMS group (Fig. 1F). The obtained results indicated that GRb1 was able to alleviate depressive-like behaviors in CMS-exposed mice.

### GRb1 promotes microglia M2-like polarization in the hippocampus of CMS-exposed mice

**Fig. 2:**
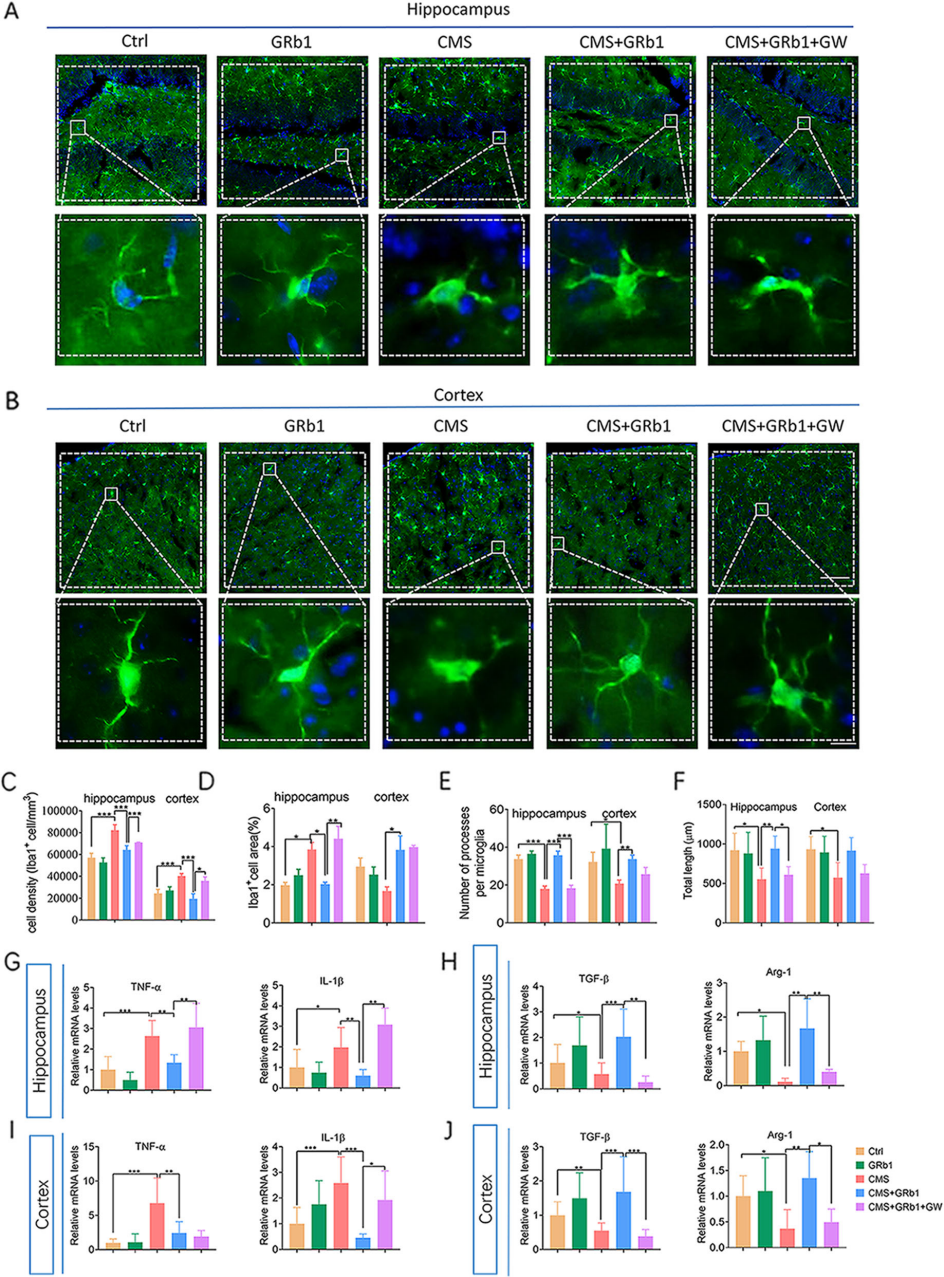
GRb1 reverses the effects of CMS on microglial phenotype and cytokine profile in mice. (**A**, **B**) Iba1^+^ cells in hippocampus and cortex were quantified by immunofluorescence. Microglial morphology was quantified by cell density (**C**), cell area (**D**), the number of processes per cell (**E**), and total length of processes per cell (**F**) (*n* = 6 mice/group). (**G**) Levels of releasing pro-inflammatory cytokines (TNF-α, IL-1β) from microglia in hippocampus. (**H**) Levels of releasing anti-inflammatory cytokines (TGF-β, Arg-1) from microglia in hippocampus (*n* = 5 mice/group). (**I**, **J**) Changes in levels of pro- and anti-inflammatory mediators in cortex (*n* = 5 mice/group). Scale bars, 50 μm (upper row in each panel), 10 μm (lower row in each panel). The statistical results are shown in Table S3. *P < 0.05, ** *P* < 0.01, *** *P* < 0.001

Since depression is a microglial disease [41], we next explored whether GRb1 had effect on microglial polarization. Immunofluorescence labeling was performed using the microglial specific marker, Iba1 (Fig. 2A, B). We assessed the number of microglia. Results showed that CMS significantly increased the density of Iba1^+^ cells in the hippocampus and cortex, while GRb1 reversed this change (Fig. 2C). When microglia are activated, they switch from ramified to amoeboid morphology. Correspondingly, hippocampal microglia in CMS animals had larger and bigger soma and fewer and shorter processes than the Ctrl group. GRb1 treatment of CMS-exposed mice reduced the cell area (Fig. 2D) and increased the number and the total length of microglia processes (Fig. 2E, F). Whereas cortical microglia had shorter processes and fewer branches in CMS animals than those of Ctrl animals, no significant changes were observed on cell areas in the CMS group compared with the Ctrl group. GRb1 treatment increased cell area and the number of processes in the cortex compared with those of the CMS group (Fig. 2D–F). Cortical microglia are likely to enter another reactive phase involving retraction and thickening of processes in the CMS group. It is possible that different signals control different aspects of microglia activation. In addition, polarized microglia are also distinguished by their expression profiles of pro- and anti-inflammatory cytokines. Stress increased inflammatory cytokine expression including TNF-α and IL-1β in the hippocampus and cortex, while it reduced anti-inflammatory cytokine expression including TGF-β and Arg-1. GRb1 inhibited these changes (Fig. 2G–J). GRb1 alone showed no significant effect on microglial activation. The assessment of immunofluorescence labeling and cytokine indicators showed that GRb1 activated M2-like microglia in the hippocampus of CMS-exposed mice.

### GRb1 rescues neurogenesis impairment in the hippocampus of CMS-exposed mice

**Fig. 3:**
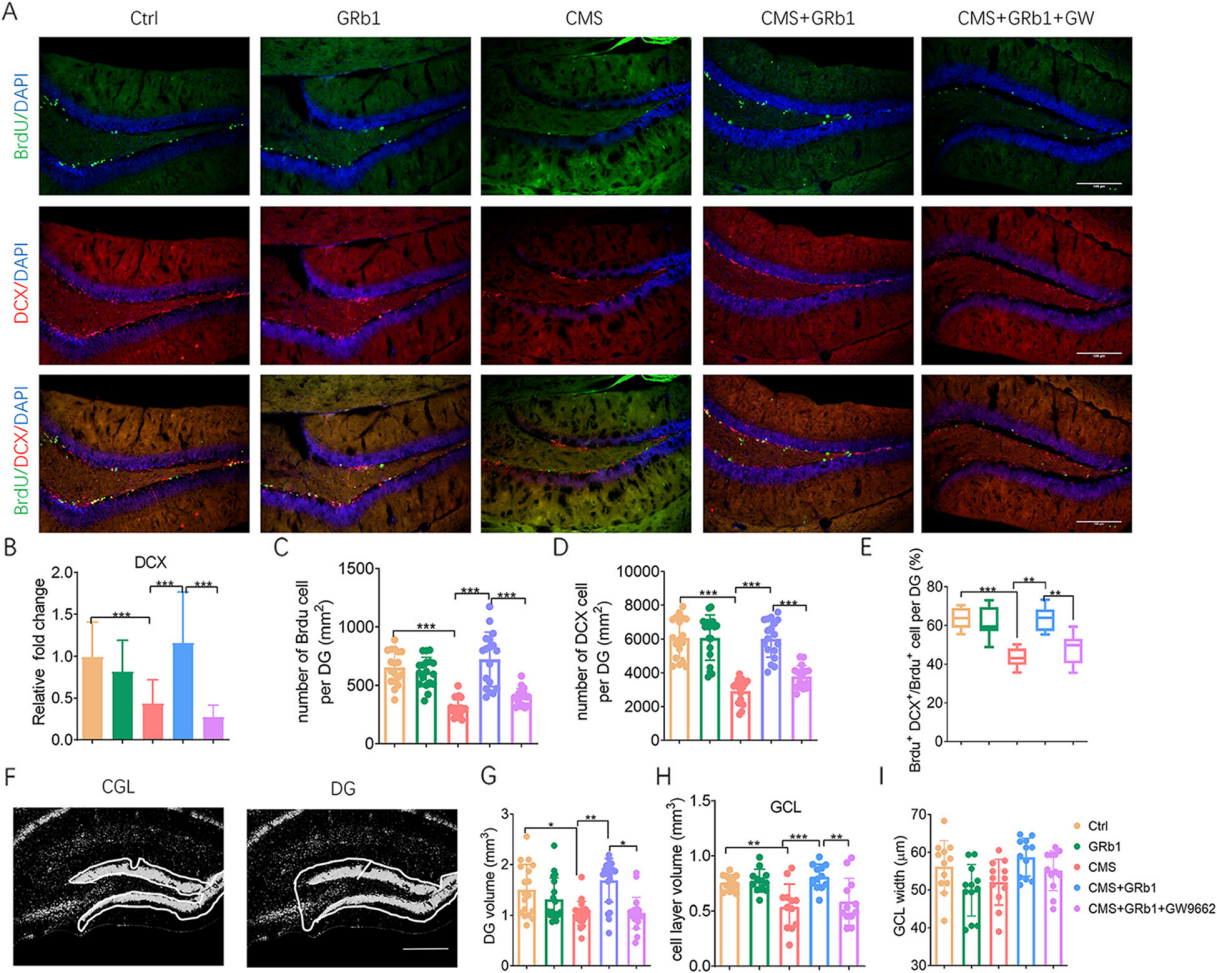
GRb1 rescues neurogenesis impairment in hippocampus of mice exposed to CMS. (**A**) Immunohistochemistry for DCX and BrdU in DG. (**B**) The mRNA levels of DCX in DG area (*n* = 5 mice/group). (**C**, **D**) Number of BrdU^+^ or DCX^+^ cells in DG area. (**E**) Differentiation ratio of proliferative cells in DG. (**F**) DAPI-stained sections of DG and GCL, which were quantified by measure DG volume (**G**), GCL volume (**H**), and GCL thickness (**I**). Scale bars: 100 μm. Data are shown as mean ± SEM (*n* = 6 mice/group). The statistical results are shown in Table S4. *P < 0.05, ** *P* < 0.01, *** *P* < 0.001

Adult hippocampal neurogenesis acts as a potential candidate mechanism for the etiology of depression [42]. We then examined whether GRb1 was involved in neurogenesis. Proliferative cells were labeled with BrdU, and newborn neurons were labeled with DCX (Fig. 3A). Chronic stress significantly decreased the mRNA level of DCX compared with control mice. Upon exposure to GRb1 treatment in the CMS group, the mRNA level of DCX increased (Fig. 3B). In addition, we observed the proliferation and differentiation of NPCs in the DG area of GRb1-treated CMS mice. The immunofluorescence results indicated that CMS decreased the number of BrdU^+^ cells in DG, which was restored by GRb1 treatment (Fig. 3C). Here, at 8 weeks post-BrdU injection, chronic stress induced a significant reduction in the number of DCX^+^ cells and the “differentiation ratio” (Brdu^+^ DCX^+^/Brdu^+^) in DG, which significantly increased in the GRb1 group (Fig. 3D, E). In order to determine the effects of GRb1 administration on the hippocampus volume, we performed an analysis of volume in DG and GCL. The results revealed that although the GCL width was not affected by CMS, the volume of the DG and GCL area was substantially reduced in the CMS group. GRb1 rescued the reduction in the volume of DG and GCL of the CMS group (Fig. 3F–I). These data indicated GRb1 promoted neurogenesis in the hippocampus of CMS-exposed mice. In contrast, no alteration was found when administering GRb1 without CMS in hippocampal neurogenesis.

### GRb1 activates PPARγ expression in CMS-induce depression mice

**Fig. 4:**
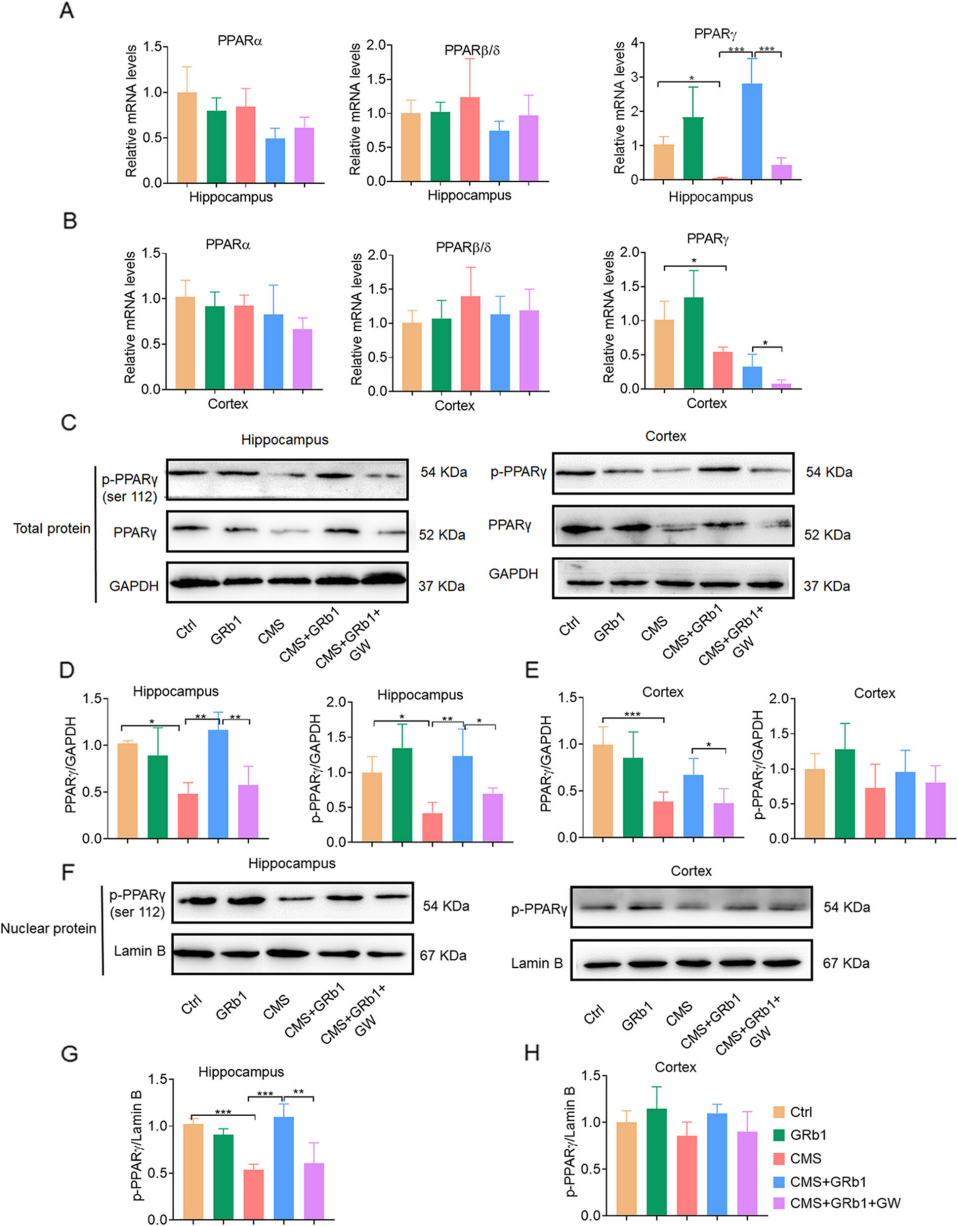
GRb1 activates PPARγ expression in CMS-induced depression model. (**A**, **B**) Relative mRNA levels of PPARа, PPARβ/δ, and PPARγ in hippocampus and cortex. (**C**, **D**) Representative western blot of p-PPARγ and PPARγ in hippocampus and cortex. (**E**, **F**) Quantification of PPARγ and p-PPARγ in hippocampus and cortex. (**G**, **H**) Representative western blot of p-PPARγ in nuclei of hippocampus and cortex. (**I**, **J**) Quantification of p-PPARγ in nuclei of hippocampus and cortex (*n* = 5 mice/group). The statistical results are shown in Table S5. *P < 0.05, ** *P* < 0.01, *** *P* < 0.001

CMS reduced mRNA expression of PPARγ, but not PPARα or PPARβ/δ, in the hippocampus and cortex (Fig. 4A, B). Similarly, protein expression of PPARγ was also reduced in the hippocampus of the CMS group. These effects of CMS were significantly reversed by GRb1 treatment in the hippocampus, which in turn were blocked by PPARγ antagonist GW9662 (Fig. 4C, D). In the cortex, GRb1 pretreatment caused no change in PPARγ of total protein in the CMS group compared with the CMS group, while GW9662 reduced PPARγ expression at the protein level (Fig. 4B–E). Activated PPARγ is known to localize in the nucleus, causing the activation or inhibition of target genes [26]. In our study, nuclear proteins in the hippocampus and cortex were extracted, and the protein levels of p-PPARγ in total protein and nucleus protein were analyzed (Fig. 4C–H). Western blot results showed that in the hippocampus, CMS steeply reduced levels of p-PPARγ in total protein and nuclear protein, which was effectively reversed by GRb1 administration (Fig. 4C–G). These groups had no difference in p-PPARγ expression at the protein level in the cortex (Fig. 4C–H). This finding suggested that GRb1 treatment increased the PPARγ activation in hippocampus of CMS-exposed mice.

### GRb1-treated microglia ameliorate depressive-like behaviors dependent on PPARγ activation

We first assessed whether the effect of antidepressants of GRb1-treated microglia was mediated by PPARγ activation. Mice were treated with GW9662 before GRb1 administration on CMS condition. We found that GW9662 treatment notably blocked the effects of antidepressants of GRb1 on the decrease of body weight, the increase of the immobility time in FST, and the reduction of sucrose consumption, without affecting autonomic activity. The GW9662 administration also caused similar trends in both weight change and sucrose preference as those in the CMS group at week 4 and week 8 (Fig. 1B–F). We then examined the interception of GW9662 administration on GRb1-treated microglial activation. Indeed, GW9662 administration weakened the effects of GRb1 on Iba1^+^ cell number and cell area (Fig. 2C, D) and microglial process in the hippocampus (Fig. 2E, F). In the cortex, the GW9662 treatment blocked only the effects of GRb1 on Iba1^+^ cell number in CMS-exposed mice (Fig. 2C). We also found that GW9662 treatment dramatically reversed GRb1-treated downregulation of pro-inflammatory cytokines and upregulation of anti-inflammatory cytokines in hippocampus (Fig. 2G, H). Similar results were observed in the cortex, except that GW9662 treatment did not affect GRb1-treated downregulation of TNF-α (Fig. 2I, J). We then examined PPARγ inhibitor in neurogenesis in CMS mice. The GW9662 treatment dramatically decreased the mRNA level of DCX (Fig. 3B), and reduced the proliferation and differentiation of NPCs when compared with that of GRb1 administration in the CMS group (Fig. 3C–E). We also investigated the effect of GW9662 on hippocampal volume. Our data indicated that GW9662 treatment did not influence the width of GCL, but led to a decrease in the volume of DG and GCL compared with GRb1 treatment exposed to CMS mice (Fig. 3F–I). These results provided evidence that GRb1 activated pro-neurogenic microglia and promoted neurogenesis dependent on the PPARγ pathway.

### PPARγ activation mediates effects of GRb1-treated microglia in vitro

**Fig. 5:**
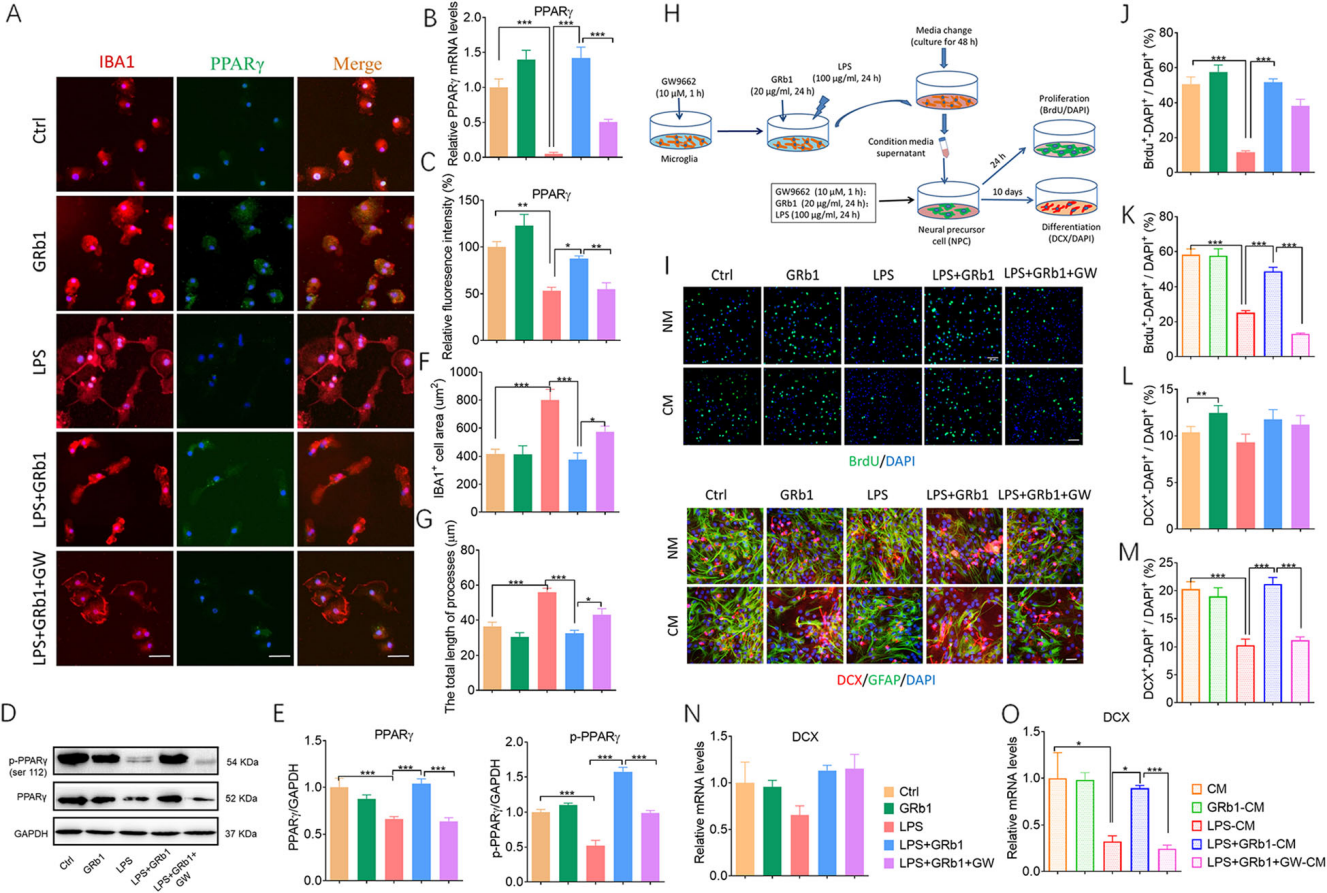
PPARγ activation mediates effects of GRb1-treated microglia in vitro. (**A**) Representative micrographs after immunostaining against Iba1 display microglial activation phenotype. Fluorescence intensity reflects PPARγ expression. Scale bars: 20 μm. (**B**) Quantification of expression of PPARγ in mRNA levels. (**C**) Quantitation of PPARγ fluorescence intensity. (**D**) Representative western blot of of PPARγ and p-PPARγ protein expression. (**E**) Quantification of PPARγ and p-PPARγ protein (*n* = 5). (**F**) The unbiased stereological quantification of microglial cell area, and (**G**) total length of processes. (**H**) Experimental protocol to test the effects of conditioned medium from microglial cultures on NPCs. (**I**) NPC cultures were maintained in native medium (NM) and directly stimulated by GRb1, LPS, and GW9662; or they were maintained in conditioned medium (CM) of activated microglia. After 2 h or 10 days, the proliferation and differentiation of NPCs were tested, respectively, by immunolabeling BrdU (upper two rows) or DCX and GFAP (lower two rows). Scale bars: 15 μm. (**J**, **K**) The ratio of BrdU^+^-DAPI^+^ to DAPI^+^ cells in NPC cultures maintained in native medium or exposed to conditioned medium from microglial cultures. (**L**, **M**) The ratio of DCX^+^-DAPI^+^ to DAPI^+^ cells in NPC cultures maintained in native medium or exposed to conditioned medium. (**N**, **O**) Relative mRNA levels of DCX in treated groups (*n* = 6). The statistical results are shown in Table S8. *P < 0.05, ** *P* < 0.01, *** *P* < 0.001

To maximize drug effectiveness in vitro, 20 μg/ml of GRb1 was chosen to be used in the following experiments to determine the regulatory effect of GRb1 on the activation of microglia and neurogenesis (Fig. S2, Table S6). Cultures were analyzed by immunofluorescence to assess microglial morphology and PPARγ expression (Fig. 5A). GRb1 could increase the mRNA level of PPARγ in the LPS group, which was reversed by GW9662 treatment (Fig. 5B). Immunofluorescence quantification showed that GRb1 pre-treatment promoted PPARγ expression in the LPS group (Fig. 5C). Moreover, GRb1 increased PPARγ and p-PPARγ protein expression by LPS treatment, while GW9662 inhibited PPARγ activation (Fig. 5D, E). Immunofluorescence was used to detect the effects of GRb1 on microglial activation. LPS-treated primary microglia exhibited round soma and developed longer processes. GRb1 pretreatment inhibited LPS-induced microglial activation, decreasing the Iba1^+^ cell area and length of processes, which was significantly suppressed by GW9662 (Fig. 5F, G). To correlate these effects with neurogenesis, we directly treated NPCs with some stimuli or conditioned medium of microglia (Fig. 5H). Proliferative cells were labeled with BrdU, and newborn neurons were labeled with DCX (Fig. 5I). GRb1 increased the ratio of BrdU^+^-DAPI^+^/DAPI^+^ cells, and GW9662 did not block these changes (Fig. 5J). In the NPC culture system, there was no significant difference in the ratio of DCX^+^-DAPI^+^ to DAPI^+^ cells between the LPS and Ctrl groups (Fig. 5L), and there were no significant changes in the mRNA and protein levels of DCX (Fig. 5N and Fig. S3A-B, Table S7). These data suggested that without microglia, LPS did not directly inhibit the differentiation of NPCs in vitro. Next, we asked whether the effects of GRb1 on neurogenesis were mediated by microglia. Primary cultures of NPCs were incubated in conditioned medium from microglia. Conditioned medium from LPS-stimuli microglia in NPCs reduced the ratios of BrdU+-DAPI+ to DAPI+ cells and DCX+-DAPI+ to DAPI+ cells, and GRb1-CM increased the ratios, whereas the effect was inhibited by pretreatment with GW9662 (Fig. 5K–M). In addition, the stimulus of microglia with GRb1 increased mRNA and protein level of DCX in the LPS-treated conditioned medium group, while pre-treatment with GW9962 significantly reversed the expression of DCX (Fig. 5O and Fig. S3C). The GRb1 alone showed no change in the ratio of BrdU+-DAPI+ to DAPI+ NPCs. Despite the slight increase in the ratio of DCX+-DAPI+ to DAPI+ NPCs observed in the GRb1 group, this relative change trend in cell numbers did not occur at the mRNA and protein levels of DCX. These results indicated that GRb1-treated pro-neurogenic microglia promoted the proliferation and differentiation of NPCs depended on PPARγ activation in vitro.

## Discussion

GRb1 shows antidepressant effects in rodent models of stress-induced depression. Our present study discloses that GRb1 alleviates depressive-like behaviors by regulating inflammatory cytokines in the hippocampus and cortex of CMS-exposed mice. Moreover, the results reveal that GRb1 treatment shifts microglia to M2-like phenotype and stimulating neurogenesis in the hippocampus. The data from in vivo and in vitro both indicates that GRb1-treated shift in pro-neurogenic microglial phenotype is dependent on the PPARγ pathway. These findings provide the first evidence that GRb1 induces a pro-neurogenic microglial phenotype via PPARγ activation in the hippocampus of CMS-exposed mice (Tables S1, S6, and S7).

In order to prove the antidepressant effect of GRb1, we conducted in vivo studies using male mice exposed to CMS. CMS model is widely used to investigate the underlying mechanism of animal lines of depression [43]. The most obvious feature of CMS is anhedonia, which is a decreased preference for sugar water. Another feature is an extinction-like inhibitory learning behavior, which is a longer immobility time in both FST and TST. GRb1 and IMI administration could reverse the effects of anhedonia and desperate behaviors. Since no alterations in the spontaneous locomotor activity were found, the results indicated that GRb1 exerted antidepressant effects in the CMS depression model and did not affect the locomotor ability. There were previous reports that GRb1 ameliorated depressive-like behaviors in ovariectomized female mice [44, 45], which indicated that female sex hormones were implicated in the pathogenesis of MDD and antidepressant responses. We analyzed only male mice in order to remove sex as a potential confounder from our study. Sex is an important biological variable from basic and preclinical research [46]. So the potential function of sex hormone effects of GRb1 on depression would be achieved further investigation. Our results confirmed that GRb1 exerted antidepressant effects in the stress-induced animal model of depression.

Accumulating evidence supported an association between depression and inflammatory processes. As a primary source of inflammatory molecules in the CNS, microglia showed a spectrum of polarization phenotypes responsible for the balance of pro- and anti-inflammatory mediators [47]. Our results revealed that for chronic stress-activated hippocampal microglia, microglia tended to show M1-like phenotype, causing soma enlargement, shortening, and thickening of processes. M1-like polarization of microglia was also characterized by the increasing expression of pro-inflammatory cytokines and decreasing expression of anti-inflammatory cytokines. GRb1 treatment in vivo significantly reduced the area of hippocampal Iba1^+^ cells and increased the number and total length of microglial processes. Meanwhile, GRb1 decreased the release of pro-inflammatory cytokines and increased the release of anti-inflammatory cytokines. There was no change in the area of Iba1^+^ cells in the cortex of CMS-exposed mice, and GRb1 treatment had no effect on the process length of cortical microglia. The partial inhibitory effect of GRb1 on cortical-activated microglia may be due to the sub-dose of GRb1 and different molecular mechanisms, which is in line with the reported effect of antidepressants in the hippocampus, not in the cortex [48]. The regional heterogeneity may underlie the different results observed in microglial activation parameters analyzed in the hippocampus and cortex. In addition, the cortex is associated with circuit-level connectivity abnormalities in MDD [49]. These results suggested that GRb1 altered the phenotype of microglia from pro-inflammation to anti-inflammation in CMS-exposed male mice. Noteworthily, we used whole hippocampal and cortical samples, rather than microglia isolated from mice brain for analysis of cytokine changes in mRNA level. These cytokines could be partially produced by other cells, such as neurons and astrocytes [50]. Thus, to directly detect the change of cytokine expression in M1-like or M2-like microglia, isolation microglia from brain tissue for flow cytometry and image analysis would be useful for future studies. Since there were interactions between various cell types in the brain, the direct effect of GRb1 on microglial morphology was further tested on primary microglia. The result showed that GRb1 directly prevented LPS-induced effects on M1-like microglial activation, consistent with the effects of GRb1 in vivo.

Impairment of hippocampal neurogenesis has been associated with inflammation-mediated depressive-like symptoms. Neurogenesis occurs predominantly in the SGZ of the hippocampus and subependymal ventricular zone. Several studies using magnetic resonance imaging showed similar total brain volume but smaller hippocampal volume in patients with depression than in controls [51]. Post mortem analysis of depressed patients showed a reduction in hippocampal volume [52]. Consistent with these results, our data showed that although the width of GCL showed no significant changes in the CMS-treated group, stress decreased the volume of DG and GCL. GRb1 increased the number of BrdU^+^ and DCX^+^ cells, as well as the differentiation ratio in SGZ. In vivo imaging study showed hippocampal shrinkage in patients with depression, which was likely associated with suppressed adult neurogenesis and neuronal death [53]. Previous studies have reported that persistent production of pro-inflammatory cytokines was detrimental to neurogenesis [54]. Based on our observation that GRb1 treatment shifted microglial polarization from neurotoxic to neuroprotective activation phenotype and reversed chronic stress-induced impairment of hippocampal neurogenesis. We speculated that GRb1-treated pro-neurogenic microglia played a phenotype-associated neurogenic role.

To examine that GRb1 promoted neurogenesis by upregulating M2-like microglia polarization, we treated NPCs with the conditioned medium that was generated from microglia treated with or without GRb1 in inflammation conditions. Compared with the LPS-CM group, GRb1+LPS-CM promoted the proliferation and differentiation of NPCs. In non-conditioned media, despite finding an increase in the BrdU^+^ cells in the GRb1+LPS group, there were no significant changes in the number of DCX^+^ cells and the mRNA and protein levels of DCX in the LPS group compared with the Ctrl group. These results indicated that pro-neurogenic effects of GRb1 were present in microglia in vitro. Although the use of LPS-stimulated primary microglia and conditioned medium to study the pro-neurogenic effect of microglia has limitations, this approach has allowed us to elucidate the molecular mechanism of GRb1-treated microglia in a straightforward way. The protective mechanisms of GRb1 in vitro were involved in inhibition of microglia-activated release of nitric oxide, superoxide, and TNF-α expression [55]. Other studies indicated that GRb1 in vitro treatment inhibited microglial apoptosis and upregulated the expression of brain-derived neurotrophic factor (BDNF), which exerted neuroprotective effects [56]. In our study, GRb1 enhanced neurogenesis via switching microglial phenotype from M1-like phenotype to M2-like phenotype and decreased the expression of pro-inflammatory cytokines and increased the expression of anti-inflammatory molecules.

Mounting evidence has been presented demonstrating that GRb1 has anti-inflammatory and neuroprotective effects. Researchers indicated that GRb1 elevated neural acetylcholine levels or mediated serotonergic, noradrenergic and dopaminergic systems for alleviation of depression [21, 57]. PPARs, as important nuclear transcription factors, are identified three subtypes: PPARα, PPARγ, and PPARβ/δ, which are all involved in the regulation of inflammatory conditions. However, the current study found that PPARγ could attenuate the inflammatory response in the CNS [24]. As expected, we found that CMS reduced mRNA and protein levels of PPARγ, but not those of PPARα or PPARβ/δ in the hippocampus and cortex. PPARγ activation form binds to the DNA-specific PPAR response element and regulates the transcription of its target genes. Our results showed that the activation of PPARγ regulated the expression of inflammatory cytokines and microglial phenotypes. We thus reasoned that GRb1-treated microglia may be related to PPARγ pathway. It is well established that activated PPARγ is localized in the nucleus and causes activation or repression of target genes [26]. Here, we found that p-PPARγ in nuclear protein significantly increased in hippocampus of GRb1treatment in CMS group. Consistent with our hypothesis, GRb1 could upregulate PPARγ expression, and as a result, attenuated hippocampal neurogenesis abnormalities and improved depressive-like behaviors in mice model of depression. In cortex, GRb1 treatment did not prevent this reduction in the mRNA and protein levels of PPARγ and p-PPARγ. This might be due to the fact that cortical areas are mainly involved in the disruption of neural circuits in the pathogenesis of depression [58]. After GRb1 treatment in CMS group, p-PPARγ was not completely inhibited probably due to partial activation of microglia by GRb1 through other pathways. For example, GRb1 treatment inhibited microglial apoptosis and upregulated the expression of neurotrophic factor [56]. Wang et al. showed that GRb1 took antidepressant effect through the BDNF-Trkb-CREB signaling pathway [31]. Further studies were needed to clarify these points. Our in vitro results demonstrated GRb1 remarkably shifted microglial polarization and mediated inflammatory cytokines induced by LPS in PPARγ-dependent manner.

GRb1 also altered transition of microglial phenotype, increasing neurogenesis in hippocampus. As an approach to corroborate these findings, we used PPARγ antagonist GW9662 to interfere microglial activation. The results indicated that GW9662 treatment strongly prevented GRb1-treated microglial activation in vivo and in vitro. Furthermore, the cultured NPCs using conditioned medium from microglia showed that GRb1 enhanced the proliferation and differentiation of NPCs via activating microglia in PPARγ dependent manner. The activity of PPARγ was finely regulated by various transcription factors. Recent studies have shown that the signal transducer and activator of transcription 6 (STAT6) regulated PPARγ activity, and inhibited NF-κb and STAT3 activity in M2 phenotype microglia [59]. Other studies revealed that GRb1 mediated microRNA through PPARγ signaling [60, 61]. Thus, more studies are required to explore the signaling pathway of PPARγ in GRb1-treated microglia. Our results showed that inhibition of PPARγ activation produced a significant dysregulation in neurogenesis mediated by microglia and highlighted the functional role of microglia as components of a neurogenic niche in the brain, as well as implicated the role of PPARγ activation in the proliferation and differentiation of NPCs.

## Conclusions

Our study provides strong in vivo and in vitro evidence that GRb1 exerts antidepressant effects by activating PPARγ to shift microglia towards an anti-inflammatory, pro-neurogenic phenotype. Our findings may shed light on the potential contribution of GRb1-treated microglia to promote neurogenesis as a therapeutic strategy against MDD.

## Supplementary Information

**Additional file 1: Fig. S1.** The concentration of GRb1 in hippocampus tissue was detected by LC-MS/MS technique. (a) Concentration-time peak charts of GRb1 in hippocampus. Mean concentration of GRb1 in mouse hippocampus over time in Table S1.

**Additional file 2: Fig. S2.** The effect of different dosages of GRb1 on activation of microglia in vitro. (a) Representative micrographs after immunostaining against Iba1 and PPARγ. Scale bars: 20 μm. (b–c) Relative mRNA level of pro-inflammatory cytokines (TNF-α, IL-1β) and anti-inflammatory cytokines (TGF-β, Arg-1). (d) Unbiased stereological quantification of microglial cell area, and (e) total length of processes. (f) Relative mRNA level of PPARγ. (g) Relative fluorescence intensity of PPARγ. The statistical results are shown in Table S6. *P < 0.05, ** *P* < 0.01, *** *P* < 0.001.

**Additional file 3: Fig. S3.** Activation of PPARγ increases the expression of DCX protein in GRb1-treated microglia in vitro*.* (a) Representative western blot of DCX protein expression in hippocampus and cortex (*n* = 5). (b–c) Quantification of DCX protein in cortex and hippocampus. CM, conditioned medium. The statistical results are shown in Table S7. *P < 0.05, ** *P* < 0.01, *** *P* < 0.001.

**Additional file 4: Table S1.** The concentration of GRb1 in hippocampus tissue was detected by LC-MS/MS technique in figure S1. **Table S2.** The F value and P value in multiple comparisons of Fig. 1. **Table S3.** The F value and P value in multiple comparisons of Fig. 2. **Table S4.** The F value and P value in multiple comparisons of Fig. 3. **Tablse S5.** The F value and P value in multiple comparisons of Fig. 4. **Table S6.** The F value and P value in multiple comparisons of figure S2. **Table S7.** The F value and P value in multiple comparisons of Fig. S3**Table S8.** The F value and P value in multiple comparisons of Fig. 5.

## Data Availability

All data generated and materials supporting the conclusion of the study are included within the article and its supplementary information files.

## Abbreviations

Arg: Arginase
BrdU: Bromodeoxyuridine
CMS: Chronic mild stress
CNS: Central nervous system
DAPI: 4′,6-Diamidin-2-phenylindol
DG: Dentate gyrus
FST: Force swimming test
GCL: Granular cell layer
GRb1: Ginsenoside Rb1
IL: Interleukin
IMI: Imipramine
LAT: Locomotor activity test
LPS: Lipopolysaccharide
PBS: Phosphate-buffered saline
MDD: Major depressive disorder
NPC: Neural precursor cell
OFT: Open field test
PPARγ: Peroxisome proliferator-activated receptor gamma
RT-qPCR: Reverse transcription-quantitative PCR
SGZ: Subgranular zone
SPT: Sucrose preference test
TGF: Transforming growth factor
TNF: Tumor necrosis factor
TST: Tail suspension test
BDNF: Brain-derived neurotrophic factor
CM: Conditioned medium
NM: Native medium
