# Supplementary figures and images for: Ginsenoside Rb1 induces a pro-neurogenic microglial phenotype via PPARγ activation in male mice exposed to chronic mild stress
Source: J Neuroinflammation. 2021 Aug 9;18:171. doi: 10.1186/s12974-021-02185-0 (PMC8353817; doi:10.1186/s12974-021-02185-0)

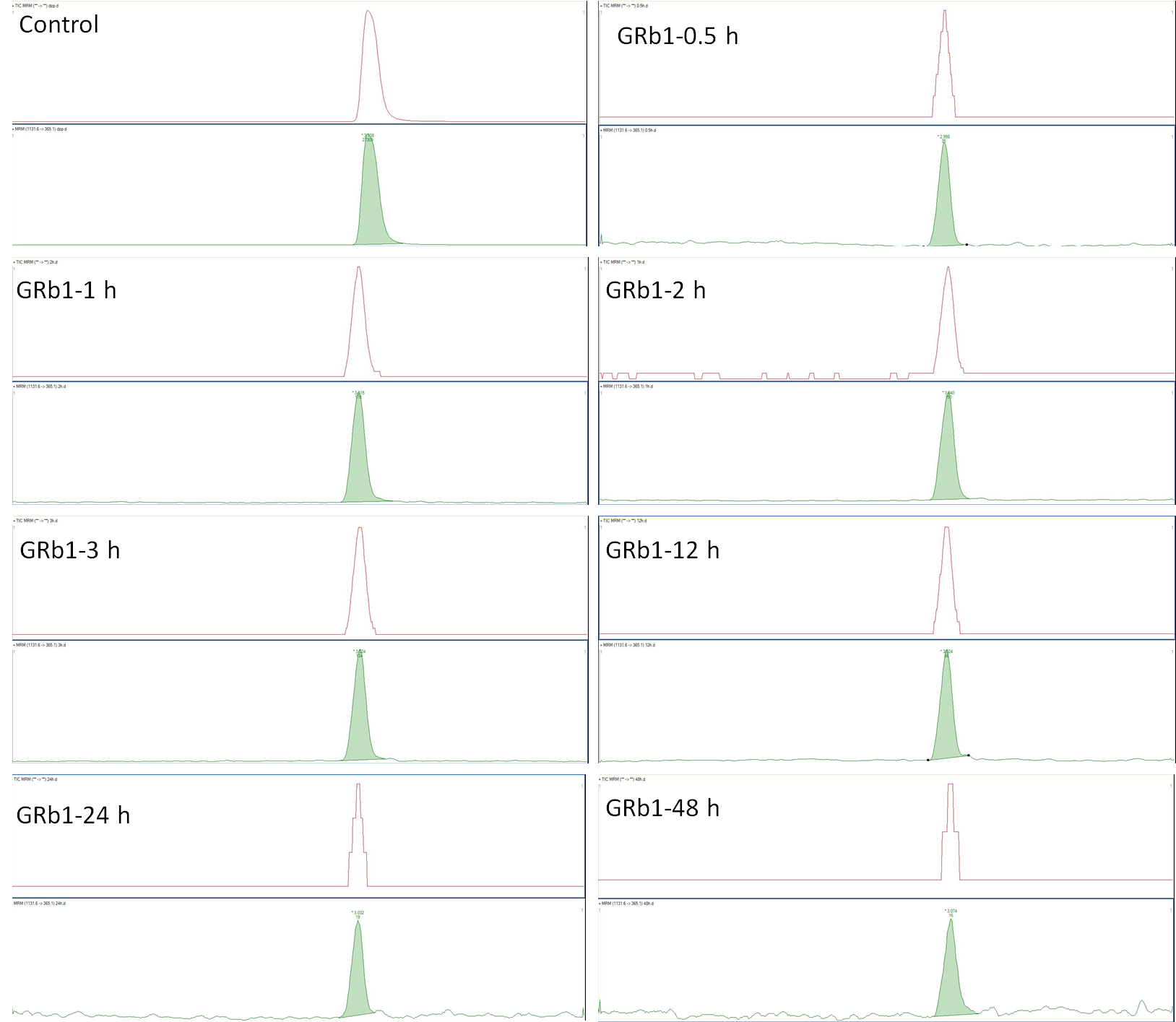

Supplement: Supplementary file 1 — Additional file 1: Fig. S1. The concentration of GRb1 in hippocampus tissue was detected by LC-MS/MS technique. (a) Concentration-time peak charts of GRb1 in hippocampus. Mean concentration of GRb1 in mouse hippocampus over time in Table S1. [file 12974_2021_2185_MOESM1_ESM.tif]

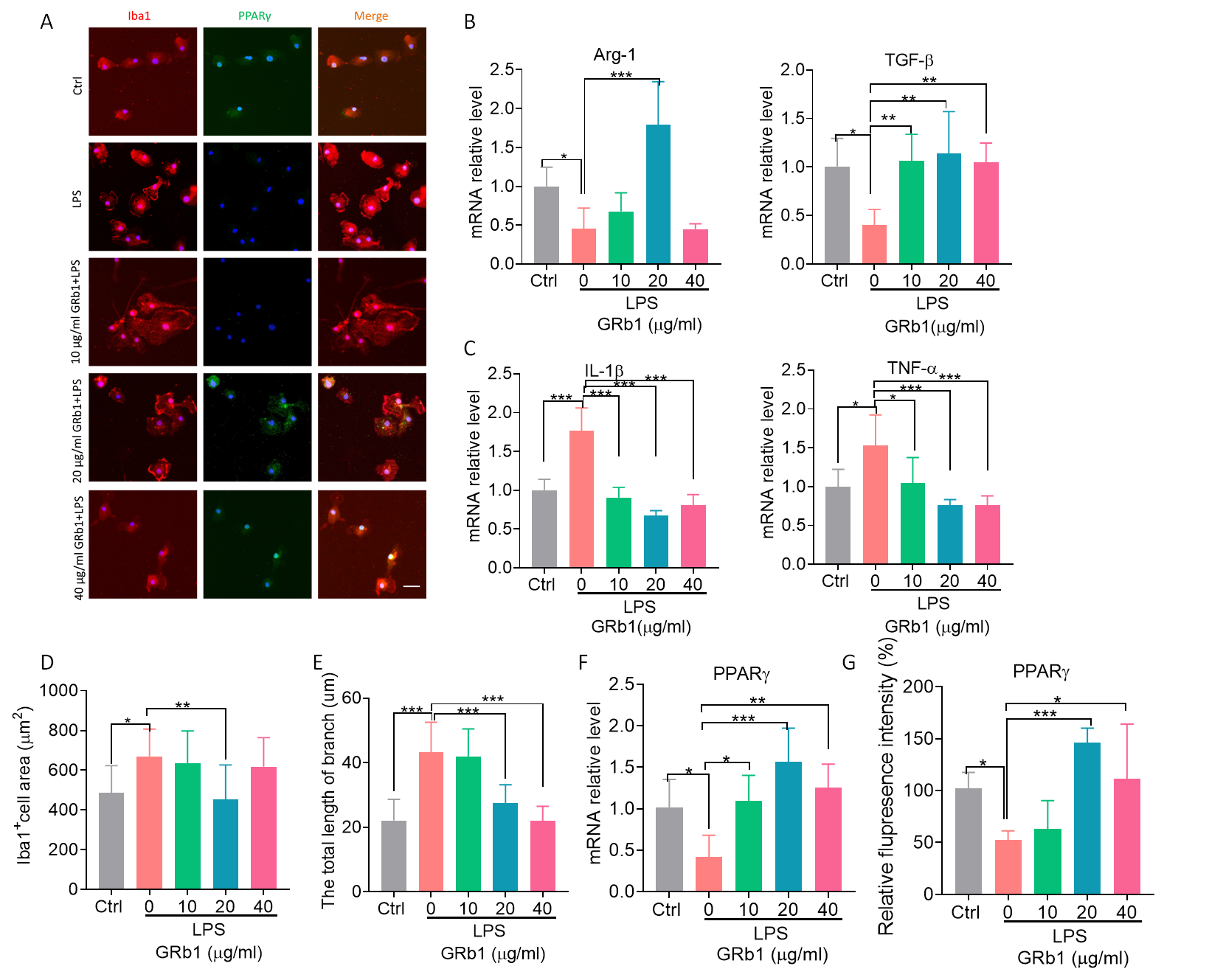

Supplement: Supplementary file 2 — Additional file 2: Fig. S2. The effect of different dosages of GRb1 on activation of microglia in vitro. (a) Representative micrographs after immunostaining against Iba1 and PPARγ. Scale bars: 20 μm. (b–c) Relative mRNA level of pro-inflammatory cytokines (TNF-α, IL-1β) and anti-inflammatory cytokines (TGF-β, Arg-1). (d) Unbiased stereological quantification of microglial cell area, and (e) total length of processes. (f) Relative mRNA level of PPARγ. (g) Relative fluorescence intensity of PPARγ. The statistical results are shown in Table S6. *P < 0.05, ** P < 0.01, *** P < 0.001. [file 12974_2021_2185_MOESM2_ESM.tif]

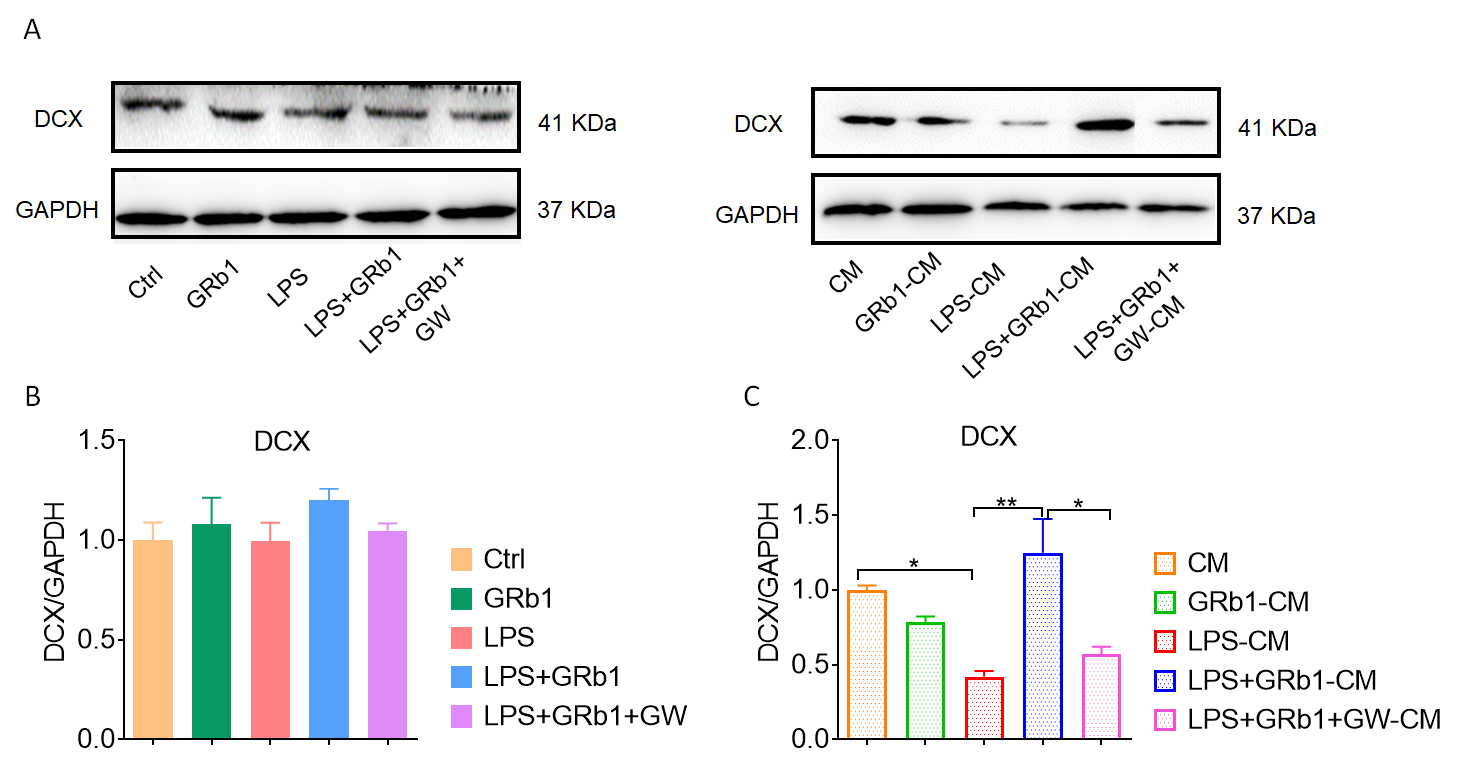

Supplement: Supplementary file 3 — Additional file 3: Fig. S3. Activation of PPARγ increases the expression of DCX protein in GRb1-treated microglia in vitro. (a) Representative western blot of DCX protein expression in hippocampus and cortex (n = 5). (b–c) Quantification of DCX protein in cortex and hippocampus. CM, conditioned medium. The statistical results are shown in Table S7. *P < 0.05, ** P < 0.01, *** P < 0.001. [file 12974_2021_2185_MOESM3_ESM.tif]
