# Supplementary material for: Ginsenoside Rb1 induces a pro-neurogenic microglial phenotype via PPARγ activation in male mice exposed to chronic mild stress
Source: J Neuroinflammation. 2021 Aug 9;18:171. doi: 10.1186/s12974-021-02185-0 (PMC8353817; doi:10.1186/s12974-021-02185-0)
Supplement: Supplementary file 4 — Additional file 4: Table S1. The concentration of GRb1 in hippocampus tissue was detected by LC-MS/MS technique in figure S1. Table S2. The F value and P value in multiple comparisons of Fig. 1. Table S3. The F value and P value in multiple comparisons of Fig. 2. Table S4. The F value and P value in multiple comparisons of Fig. 3. Tablse S5. The F value and P value in multiple comparisons of Fig. 4. Table S6. The F value and P value in multiple comparisons of figure S2. Table S7. The F value and P value in multiple comparisons of Fig. S3Table S8. The F value and P value in multiple comparisons of Fig. 5. [file 12974_2021_2185_MOESM4_ESM.zip › 12974_2021_2185_MOESM4_ESM/Table S1.docx]

**Table 6**

**Fig. S1. The concentration of GRb1 in hippocampus tissue was detected by LC-MS/MS technique.**

| **number** | **sample** | **Retention time** | **Peak area** |
| --- | --- | --- | --- |
| 1 | control | 3.108 | 27309 |
| 2 | 0.5h | 2.998 | 35 |
| 3 | 1h | 3.040 | 150 |
| 4 | 2h | 3.015 | 178 |
| 5 | 3h | 3.024 | 134 |
| 6 | 12h | 3.000 | 66 |
| 7 | 24h | 3.032 | 19 |
| 8 | 48h | 3.074 | 16 |
