# Supplementary material for: Ginsenoside Rb1 induces a pro-neurogenic microglial phenotype via PPARγ activation in male mice exposed to chronic mild stress
Source: J Neuroinflammation. 2021 Aug 9;18:171. doi: 10.1186/s12974-021-02185-0 (PMC8353817; doi:10.1186/s12974-021-02185-0)
Supplement: Supplementary file 4 — Additional file 4: Table S1. The concentration of GRb1 in hippocampus tissue was detected by LC-MS/MS technique in figure S1. Table S2. The F value and P value in multiple comparisons of Fig. 1. Table S3. The F value and P value in multiple comparisons of Fig. 2. Table S4. The F value and P value in multiple comparisons of Fig. 3. Tablse S5. The F value and P value in multiple comparisons of Fig. 4. Table S6. The F value and P value in multiple comparisons of figure S2. Table S7. The F value and P value in multiple comparisons of Fig. S3Table S8. The F value and P value in multiple comparisons of Fig. 5. [file 12974_2021_2185_MOESM4_ESM.zip › 12974_2021_2185_MOESM4_ESM/Table S2.docx]

**Table 1．The F value and P value in multiple comparisons of figure 1**

**Fig. 1. GRb1 alleviates depressive-like behaviors in male mice exposed to CMS.**

| *figure* | group | F or T | P | N |
| --- | --- | --- | --- | --- |
| *Figure 1B-1* | CMS vs. Ctrl |  | 0.0970 |  |
|  | GRb1 vs. Ctrl | 4.612 | 0.1135 | 12 |
|  | CMS+GRb1 vs. CMS |  | 0.9942 |  |
|  | CMS+IMI vs. CMS |  | 0.2171 |  |
|  | CMS+IMI vs. CMS+GRb1 | 1.903 | 0.0702 |  |
|  | CMS+GRb1+GW vs. CMS+GRb1 | 1.734 | 0.0968 | 12 |
| *Figure 1B-2* | CMS vs. Ctrl |  | 0.2054 |  |
|  | GRb1 vs. Ctrl | 1.411 | 0.6570 | 12 |
|  | CMS+ GRb1 vs. CMS |  | 0.6361 |  |
|  | CMS+IMI vs. CMS |  | 0.9489 |  |
|  | CMS+ IMI vs. CMS+GRb1 | 0.076 | 0.9399 |  |
|  | CMS+GRb1+GW vs. CMS+GRb1 | 0.336 | 0.7401 | 12 |
|  | Treatment vs Basal (Ctrl) |  | 0.0020 |  |
| *Figure 1C-1* | Treatment vs Basal (GRb1) |  | 0.0010 |  |
|  | Treatment vs Basal (CMS) |  | 0.0750 |  |
|  | Treatment vs Basal (CMS+GRb1) | 14.211 | < 0.0001 | 12 |
|  | Treatment vs Basal (CMS+IMI) |  | < 0.0001 |  |
|  | Treatment vs Basal (CMS+GRb1+GW) |  | 0.0960 |  |
|  | Ctrl vs CMS (Basal) |  | < 0.0001 |  |
|  | CMS+GRb1 vs CMS (treatment) |  | < 0.0001 |  |
|  | CMS+IMI vs CMS (treatment) | 4.540 | 0.0163 | 12 |
|  | CMS+GRb1+GW vs. CMS+GRb1 (treatment) |  | < 0.0001 |  |
| *Figure 1C-2* | CMS vs. Ctrl |  | 0.0343 |  |
|  | GRb1 vs. Ctrl | 4.636 | 0.9032 | 12 |
|  | CMS+ GRb1 vs. CMS |  | 0.0035 |  |
|  | CMS+IMI vs. CMS |  | 0.1519 |  |
|  | CMS+ IMI vs. CMS+GRb1 | 0.472 | 0.6416 |  |
|  | CMS+GRb1+GW vs. CMS+GRb1 | 4.255 | 0.0003 | 12 |
| *Figure 1D* | CMS vs. Ctrl |  | 0.0002 |  |
|  | GRb1 vs. Ctrl | 43.820 | >0.9999 | 12 |
|  | CMS+ GRb1 vs. CMS |  | <0.0001 |  |
|  | CMS+IMI vs. CMS |  | 0.0006 |  |
|  | CMS+ IMI vs. CMS+GRb1 | 1.097 | 0.2846 |  |
|  | CMS+GRb1+GW vs. CMS+GRb1 | 4.721 | 0.0001 | 12 |
| *Figure 1E* | CMS vs. Ctrl |  | < 0.0001 |  |
|  | GRb1 vs. Ctrl | 6.458 | 0.6054 | 12 |
|  | CMS+GRb1 vs. CMS |  | < 0.0001 |  |
|  | CMS+IMI vs. CMS |  | 0.0001 |  |
|  | CMS+ IMI vs. CMS+GRb1 | 0.513 | 0.6127 |  |
|  | CMS+GRb1+GW vs. CMS+GRb1 | 4.228 | 0.0003 | 12 |

|  | Treatment vs Basal (Ctrl) |  | 0.0720 |  |
| --- | --- | --- | --- | --- |
|  | Treatment vs Basal (GRb1) |  | 0.1241 |  |
| *Figure 1F-1* | Treatment vs Basal (CMS) |  | 0.4190 |  |
|  | Treatment vs Basal (CMS+GRb1) | 7.804 | < 0.0001 | 12 |
|  | Treatment vs Basal (CMS+IMI) |  | < 0.0001 |  |
|  | Treatment vs Basal (CMS+GRb1+GW) |  | 0.4515 |  |
|  | Ctrl vs CMS (Basal) |  | < 0.0001 |  |
|  | CMS+GRb1 vs CMS (treatment) |  | < 0.0001 |  |
|  | CMS+IMI vs CMS (treatment) | 18.730 | < 0.0001 | 12 |
|  | CMS+GRb1+GW vs. CMS+GRb1 (treatment) |  | < 0.0001 |  |
| *Figure 1F-2* | CMS vs. Ctrl |  | < 0.0001 |  |
|  | GRb1 vs. Ctrl | 33.920 | 0.1233 | 12 |
|  | CMS+ GRb1 vs. CMS |  | < 0.0001 |  |
|  | CMS+IMI vs. CMS |  | < 0.0001 |  |
|  | CMS+ IMI vs. CMS+GRb1 | 0.809 | 0.4287 |  |
|  | CMS+GRb1+GW vs. CMS+GRb1 | 3.135 | 0.0057 | 12 |
