# Supplementary material for: Ginsenoside Rb1 induces a pro-neurogenic microglial phenotype via PPARγ activation in male mice exposed to chronic mild stress
Source: J Neuroinflammation. 2021 Aug 9;18:171. doi: 10.1186/s12974-021-02185-0 (PMC8353817; doi:10.1186/s12974-021-02185-0)
Supplement: Supplementary file 4 — Additional file 4: Table S1. The concentration of GRb1 in hippocampus tissue was detected by LC-MS/MS technique in figure S1. Table S2. The F value and P value in multiple comparisons of Fig. 1. Table S3. The F value and P value in multiple comparisons of Fig. 2. Table S4. The F value and P value in multiple comparisons of Fig. 3. Tablse S5. The F value and P value in multiple comparisons of Fig. 4. Table S6. The F value and P value in multiple comparisons of figure S2. Table S7. The F value and P value in multiple comparisons of Fig. S3Table S8. The F value and P value in multiple comparisons of Fig. 5. [file 12974_2021_2185_MOESM4_ESM.zip › 12974_2021_2185_MOESM4_ESM/Table S3.docx]

**Table 2．The F value and P value in multiple comparisons of figure 2**

**Fig. 2. GRb1 reverses the effects of CMS on microglial phenotype and cytokine profile in mice.**

| *figure* | group | F or T | P | N |
| --- | --- | --- | --- | --- |
| *Figure 2C-h* | CMS vs. Ctrl |  | < 0.0001 |  |
|  | GRb1 vs. Ctrl | 33.920 | 0.1233 | 6 |
|  | CMS+GRb1 vs. CMS |  | < 0.0001 |  |
|  | CMS+GRb1+GW vs. CMS+GRb1 | 8.648 | < 0.0001 | 6 |
| *Figure 2C-c* | CMS vs. Ctrl |  | 0.0002 |  |
|  | GRb1 vs. Ctrl | 7.923 | 0.2627 | 6 |
|  | CMS+ GRb1 vs. CMS |  | 0.0004 |  |
|  | CMS+GRb1+GW vs. CMS+GRb1 | 2.444 | 0.0193 | 6 |
| *Figure 2D-h* | CMS vs. Ctrl |  | 0.0207 |  |
|  | GRb1 vs. Ctrl | 2.347 | 0.6339 | 5 |
|  | CMS+ GRb1 vs. CMS |  | 0.0155 |  |
|  | CMS+GRb1+GW vs. CMS+GRb1 | 3.191 | 0.0057 | 6 |
| *Figure 2D-c* | CMS vs. Ctrl |  | 0.2343 |  |
|  | GRb1 vs. Ctrl | 8.559 | 0.2388 | 6 |
|  | CMS+GRb1 vs. CMS |  | 0.0441 |  |
|  | CMS+GRb1+GW vs. CMS+GRb1 | 2.107 | 0.0682 |  |
| *Figure 2E-h* | CMS vs. Ctrl |  | 0.0006 |  |
|  | GRb1 vs. Ctrl | 5.422 | 0.1039 | 6 |
|  | CMS+GRb1 vs. CMS |  | 0.0001 |  |
|  | CMS+GRb1+GW vs. CMS+GRb1 | 6.878 | < 0.0001 |  |
| *Figure 2E-c* | CMS vs. Ctrl |  | 0.0169 |  |
|  | GRb1 vs. Ctrl | 1.380 | 0.0732 | 6 |
|  | CMS+GRb1 vs. CMS |  | 0.0056 |  |
|  | CMS+GRb1+GW vs. CMS+GRb1 | 0.445 | 0.6611 |  |

| *Figure 2F-h* | CMS vs. Ctrl |  | 0.0142 |  |
| --- | --- | --- | --- | --- |
|  | GRb1 vs. Ctrl | 1.530 | 0.3609 | 6 |
|  | CMS+GRb1 vs. CMS |  | 0.0017 |  |
|  | CMS+GRb1+GW vs. CMS+GRb1 | 2.139 | 0.0482 |  |
| *Figure 2F-c* | CMS vs. Ctrl |  | 0.0151 |  |
|  | GRb1 vs. Ctrl | 1.862 | 0.4787 | 6 |
|  | CMS+GRb1 vs. CMS |  | 0.9884 |  |
|  | CMS+GRb1+GW vs. CMS+GRb1 | 0.933 | 0.3783 |  |
| *Figure 2G-1* | CMS vs. Ctrl |  | <0.0001 |  |
|  | GRb1 vs. Ctrl | 1.443 | 0.7468 | 5 |
|  | CMS+GRb1 vs. CMS |  | 0.0057 |  |
|  | CMS+GRb1+GW vs. CMS+GRb1 | 4.834 | 0.0013 | 5 |
| *Figure 2G-2* | CMS vs. Ctrl |  | 0.0329 |  |
|  | GRb1 vs. Ctrl | 3.340 | 0.8778 | 5 |
|  | CMS+GRb1 vs. CMS |  | 0.0015 |  |
|  | CMS+GRb1+GW vs. CMS+GRb1 | 3.610 | 0.0069 | 5 |
| *Figure 2H-1* | CMS vs. Ctrl |  | 0.0109 |  |
|  | GRb1 vs. Ctrl | 9.361 | 0.8942 | 5 |
|  | CMS+GRb1 vs. CMS |  | 0.0004 |  |
|  | CMS+GRb1+GW vs. CMS+GRb1 | 4.258 | 0.0028 | 5 |
| *Figure 2H-2* | CMS vs. Ctrl |  | 0.0159 |  |
|  | GRb1 vs. Ctrl | 6.216 | 0.6888 | 5 |
|  | CMS+GRb1 vs. CMS |  | 0.0011 |  |
|  | CMS+GRb1+GW vs. CMS+GRb1 | 4.040 | 0.0037 | 5 |
| *Figure 2I-1* | CMS vs. Ctrl |  | 0.0003 |  |
|  | GRb1 vs. Ctrl | 6.799 | 0.9950 | 5 |
|  | CMS+GRb1 vs. CMS |  | 0.0048 |  |
|  | CMS+GRb1+GW vs. CMS+GRb1 | 1.464 | 0.1812 | 5 |
| *Figure 2I-2* | CMS vs. Ctrl |  | 0.0005 |  |
|  | GRb1 vs. Ctrl | 30.290 | 0.2315 | 5 |
|  | CMS+GRb1 vs. CMS |  | < 0.0001 |  |
|  | CMS+GRb1+GW vs. CMS+GRb1 | 2.646 | 0.0294 | 5 |
| *Figure 2J-1* | CMS vs. Ctrl |  | 0.0073 |  |
|  | GRb1 vs. Ctrl | 11.820 | 0.9568 | 5 |
|  | CMS+GRb1 vs. CMS |  | 0.0002 |  |
|  | CMS +GRb1+GW vs. CMS+GRb1 | 7.331 | < 0.0001 | 5 |
| *Figure 2J-2* | CMS vs. Ctrl |  | 0.0282 |  |
|  | GRb1 vs. Ctrl | 16.090 | 0.3674 | 5 |
|  | CMS+GRb1 vs. CMS |  | 0.0010 |  |
|  | CMS+GRb1+GW vs. CMS+GRb1 | 3.215 | 0.0123 | 5 |

h, hippocampus; c, cortex
