# Supplementary material for: Ginsenoside Rb1 induces a pro-neurogenic microglial phenotype via PPARγ activation in male mice exposed to chronic mild stress
Source: J Neuroinflammation. 2021 Aug 9;18:171. doi: 10.1186/s12974-021-02185-0 (PMC8353817; doi:10.1186/s12974-021-02185-0)
Supplement: Supplementary file 4 — Additional file 4: Table S1. The concentration of GRb1 in hippocampus tissue was detected by LC-MS/MS technique in figure S1. Table S2. The F value and P value in multiple comparisons of Fig. 1. Table S3. The F value and P value in multiple comparisons of Fig. 2. Table S4. The F value and P value in multiple comparisons of Fig. 3. Tablse S5. The F value and P value in multiple comparisons of Fig. 4. Table S6. The F value and P value in multiple comparisons of figure S2. Table S7. The F value and P value in multiple comparisons of Fig. S3Table S8. The F value and P value in multiple comparisons of Fig. 5. [file 12974_2021_2185_MOESM4_ESM.zip › 12974_2021_2185_MOESM4_ESM/Table S4.docx]

**Table 3．The F value and P value in multiple comparisons of figure 3**

**Fig. 3. GRb1 rescues neurogenesis impairment in hippocampus of mice exposed to CMS.**

| *figure* | group | F or T | P | N |
| --- | --- | --- | --- | --- |
| *Figure 3B* | CMS vs. Ctrl |  | 0.0008 |  |
|  | GRb1 vs. Ctrl | 27.880 | 0.2877 | 5 |
|  | CMS+GRb1 vs. CMS |  | < 0.0001 |  |
|  | CMS+GRb1+GW vs. CMS+GRb1 | 7.291 | < 0.0001 |  |
| *Figure 3C* | CMS vs. Ctrl |  | < 0.0001 |  |
|  | GRb1 vs. Ctrl | 47.020 | 0.7149 | 6 |
|  | CMS+GRb1 vs. CMS |  | < 0.0001 |  |
|  | CMS+GRb1+GW vs. CMS+GRb1 | 8.922 | < 0.0001 |  |
| *Figure 3D* | CMS vs. Ctrl |  | < 0.0001 |  |
|  | GRb1 vs. Ctrl | 30.200 | 0.9995 | 6 |
|  | CMS+GRb1 vs. CMS |  | < 0.0001 |  |
|  | CMS+GRb1+GW vs. CMS+GRb1 | 6.145 | 0.0003 |  |
| *Figure 3E* | CMS vs. Ctrl |  | < 0.0001 |  |
|  | GRb1 vs. Ctrl | 4.820 | 0.9971 | 6 |
|  | CMS+GRb1 vs. CMS |  | 0.0094 |  |
|  | CMS+GRb1+GW vs. CMS+GRb1 | 3.659 | 0.0064 |  |
| *Figure 3G* | CMS vs. Ctrl |  | 0.0399 |  |
|  | GRb1 vs. Ctrl | 10.990 | 0.4946 | 6 |
|  | CMS+GRb1 vs. CMS |  | 0.0021 |  |
|  | CMS+GRb1+GW vs. CMS+GRb1 | 2.858 | 0.0212 |  |
| *Figure 3H* | CMS vs. Ctrl |  | 0.0050 |  |
|  | GRb1 vs. Ctrl | 1.572 | 0.9985 | 6 |
|  | CMS+GRb1 vs. CMS |  | 0.0003 |  |
|  | CMS+GRb1+GW vs. CMS+GRb1 | 3.266 | 0.0035 |  |
| *Figure 3I* | CMS vs. Ctrl |  | 0.5433 |  |
|  | GRb1 vs. Ctrl | 0.6268 | 0.1561 | 6 |
|  | CMS+GRb1 vs. CMS |  | 0.0584 |  |
|  | CMS+GRb1+GW vs. CMS+GRb1 | 1.703 | 0.1027 |  |
