# Supplementary material for: Ginsenoside Rb1 induces a pro-neurogenic microglial phenotype via PPARγ activation in male mice exposed to chronic mild stress
Source: J Neuroinflammation. 2021 Aug 9;18:171. doi: 10.1186/s12974-021-02185-0 (PMC8353817; doi:10.1186/s12974-021-02185-0)
Supplement: Supplementary file 4 — Additional file 4: Table S1. The concentration of GRb1 in hippocampus tissue was detected by LC-MS/MS technique in figure S1. Table S2. The F value and P value in multiple comparisons of Fig. 1. Table S3. The F value and P value in multiple comparisons of Fig. 2. Table S4. The F value and P value in multiple comparisons of Fig. 3. Tablse S5. The F value and P value in multiple comparisons of Fig. 4. Table S6. The F value and P value in multiple comparisons of figure S2. Table S7. The F value and P value in multiple comparisons of Fig. S3Table S8. The F value and P value in multiple comparisons of Fig. 5. [file 12974_2021_2185_MOESM4_ESM.zip › 12974_2021_2185_MOESM4_ESM/Table S5.docx]

**Table 4．The F value and P value in multiple comparisons of figure 4**

**Fig. 4. GRb1 activates PPARγ expression in CMS-induce depression model.**

| *figure* | group | F or T | P | N |
| --- | --- | --- | --- | --- |
| *Figure 4A-1* | CMS vs. Ctrl |  | 0.6949 |  |
|  | GRb1 vs. Ctrl | 1.312 | 0.5732 | 5 |
|  | CMS+GRb1 vs. CMS |  | 0.0525 |  |
|  | CMS+GRb1+GW vs. CMS+GRb1 | 1.602 | 0.1479 | 5 |
| *Figure 4A-2* | CMS vs. Ctrl |  | 0.6263 |  |
|  | GRb1 vs. Ctrl | 1.201 | 0.9992 | 5 |
|  | CMS+GRb1 vs. CMS |  | 0.1051 |  |
|  | CMS+GRb1+GW vs. CMS+GRb1 | 1.532 | 0.1642 | 5 |
| *Figure 4A-3* | CMS vs. Ctrl |  | 0.0257 |  |
|  | GRb1 vs. Ctrl | 2.977 | 0.0790 | 5 |
|  | CMS+GRb1 vs. CMS |  | <0.0001 |  |
|  | CMS+GRb1+GW vs. CMS+GRb1 | 7.197 | <0.0001 | 5 |
| *Figure 4B-1* | CMS vs. Ctrl |  | 0.8421 |  |
|  | GRb1 vs. Ctrl | 2.376 | 0.7990 | 5 |
|  | CMS+GRb1 vs. CMS |  | 0.8167 |  |
|  | CMS+GRb1+GW vs. CMS+GRb1 | 1.019 | 0.3382 | 5 |
| *Figure 4B-2* | CMS vs. Ctrl |  | 0.2730 |  |
|  | GRb1 vs. Ctrl | 0.435 | 0.9892 | 5 |
|  | CMS+GRb1 vs. CMS |  | 0.5633 |  |
|  | CMS+GRb1+GW vs. CMS+GRb1 | 0.3501 | 0.7353 | 5 |
| *Figure 4B-3* | CMS vs. Ctrl |  | 0.0450 |  |
|  | GRb1 vs. Ctrl | 1.612 | 0.1878 | 5 |
|  | CMS+GRb1 vs. CMS |  | 0.5048 |  |
|  | CMS+GRb1+GW vs. CMS+GRb1 | 2.916 | 0.0194 | 5 |
| *Figure 4D-1* | CMS vs. Ctrl |  | 0.0119 |  |
|  | GRb1 vs. Ctrl | 9.619 | 0.9997 | 5 |
|  | CMS+GRb1 vs. CMS |  | 0.0010 |  |
|  | CMS+GRb1+GW vs. CMS+GRb1 | 4.306 | 0.0026 | 5 |
| *Figure 4D-2* | CMS vs. Ctrl |  | 0.0404 |  |
|  | GRb1 vs. Ctrl | 0.862 | 0.3027 | 5 |
|  | CMS+GRb1 vs. CMS |  | 0.0048 |  |
|  | CMS+GRb1+GW vs. CMS+GRb1 | 3.017 | 0.0166 | 5 |
| *Figure 4E-1* | CMS vs. Ctrl |  | 0.0006 |  |
|  | GRb1 vs. Ctrl | 2.227 | 0.6013 | 5 |
|  | CMS+GRb1 vs. CMS |  | 0.0939 |  |
|  | CMS+GRb1+GW vs. CMS+GRb1 | 2.829 | 0.0222 | 5 |
| *Figure 4E-2* | CMS vs. Ctrl |  | 0.3427 |  |
|  | GRb1 vs. Ctrl | 3.501 | 0.3302 | 5 |
|  | CMS+GRb1 vs. CMS |  | 0.4876 |  |
|  | CMS+GRb1+GW vs. CMS+GRb1 | 0.861 | 0.4143 | 5 |
| *Figure 4G* | CMS vs. Ctrl |  | <0.0001 |  |
|  | GRb1 vs. Ctrl | 0.888 | 0.2148 | 5 |
|  | CMS+GRb1 vs. CMS |  | <0.0001 |  |
|  | CMS+GRb1+GW vs. CMS+GRb1 | 4.299 | 0.0026 | 5 |
| *Figure 4H* | CMS vs. Ctrl |  | 0.5627 |  |
|  | GRb1 vs. Ctrl | 1.490 | 0.4920 | 5 |
|  | CMS+GRb1 vs. CMS |  | 0.9838 |  |
|  | CMS+GRb1+GW vs. CMS+GRb1 | 0.732 | 0.4853 | 5 |
