# Supplementary material for: Ginsenoside Rb1 induces a pro-neurogenic microglial phenotype via PPARγ activation in male mice exposed to chronic mild stress
Source: J Neuroinflammation. 2021 Aug 9;18:171. doi: 10.1186/s12974-021-02185-0 (PMC8353817; doi:10.1186/s12974-021-02185-0)
Supplement: Supplementary file 4 — Additional file 4: Table S1. The concentration of GRb1 in hippocampus tissue was detected by LC-MS/MS technique in figure S1. Table S2. The F value and P value in multiple comparisons of Fig. 1. Table S3. The F value and P value in multiple comparisons of Fig. 2. Table S4. The F value and P value in multiple comparisons of Fig. 3. Tablse S5. The F value and P value in multiple comparisons of Fig. 4. Table S6. The F value and P value in multiple comparisons of figure S2. Table S7. The F value and P value in multiple comparisons of Fig. S3Table S8. The F value and P value in multiple comparisons of Fig. 5. [file 12974_2021_2185_MOESM4_ESM.zip › 12974_2021_2185_MOESM4_ESM/Table S6.docx]

**Table 7．The F value and P value in multiple comparisons of figure S2**

**Fig. S2. The effect of different dosages of GRb1 on activation of microglia *in vitro*.**

| *figure* | group | F | P | N |
| --- | --- | --- | --- | --- |
| *Figure 6B-1* | LPS vs. Ctrl |  | 0.0454 |  |
|  | 10 μg/ml LPS+GRb1 vs. LPS | 18.940 | 0.4097 | 6 |
|  | 20 μg/ml LPS+GRb1 vs. LPS |  | < 0.0001 |  |
|  | 40 μg/ml LPS+GRb1 vs. LPS |  | > 0.9999 |  |
| *Figure 6B-2* | LPS vs. Ctrl |  | 0.0100 |  |
|  | 10 μg/ml LPS+GRb1 vs. LPS | 6.515 | 0.0042 | 6 |
|  | 20 μg/ml LPS+GRb1 vs. LPS |  | 0.0014 |  |
|  | 40 μg/ml LPS+GRb1 vs. LPS |  | 0.0055 |  |
| *Figure 6C-1* | LPS vs. Ctrl |  | < 0.0001 |  |
|  | 10 μg/ml LPS+GRb1 vs. LPS | 37.831 | < 0.0001 | 6 |
|  | 20 μg/ml LPS+GRb1 vs. LPS |  | < 0.0001 |  |
|  | 40 μg/ml LPS+GRb1 vs. LPS |  | < 0.0001 |  |
| *Figure 6C-2* | LPS vs. Ctrl |  | 0.0133 |  |
|  | 10 μg/ml LPS+GRb1 vs. LPS | 8.756 | 0.0258 | 6 |
|  | 20 μg/ml LPS+GRb1 vs. LPS |  | 0.0002 |  |
|  | 40 μg/ml LPS+GRb1 vs. LPS |  | 0.0002 |  |
| *Figure 6D* | LPS vs. Ctrl |  | 0.0357 |  |
|  | 10 μg/ml LPS+GRb1 vs. LPS | 4.800 | 0.9795 | 12 |
|  | 20 μg/ml LPS+GRb1 vs. LPS |  | 0.0083 |  |
|  | 40 μg/ml LPS+GRb1 vs. LPS |  | 0.9179 |  |
| *Figure 6E* | LPS vs. Ctrl |  | < 0.0001 |  |
|  | 10 μg/ml LPS+GRb1 vs. LPS | 25.740 | 0.9891 | 12 |
|  | 20 μg/ml LPS+GRb1 vs. LPS |  | < 0.0001 |  |
|  | 40 μg/ml LPS+GRb1 vs. LPS |  | < 0.0001 |  |
| *Figure 6F* | LPS vs. Ctrl |  | 0.0284 |  |
|  | 10 μg/ml LPS+GRb1 vs. LPS | 10.021 | 0.0113 | 6 |
|  | 20 μg/ml LPS+GRb1 vs. LPS |  | < 0.0001 |  |
|  | 40 μg/ml LPS+GRb1 vs. LPS |  | 0.0013 |  |
| *Figure 6G* | LPS vs. Ctrl |  | 0.0404 |  |
|  | 10 μg/ml LPS+GRb1 vs. LPS | 10.740 | 0.9650 | 10 |
|  | 20 μg/ml LPS+GRb1 vs. LPS |  | < 0.0001 |  |
|  | 40 μg/ml LPS+GRb1 vs. LPS |  | 0.0105 |  |
