# Supplementary material for: Ginsenoside Rb1 induces a pro-neurogenic microglial phenotype via PPARγ activation in male mice exposed to chronic mild stress
Source: J Neuroinflammation. 2021 Aug 9;18:171. doi: 10.1186/s12974-021-02185-0 (PMC8353817; doi:10.1186/s12974-021-02185-0)
Supplement: Supplementary file 4 — Additional file 4: Table S1. The concentration of GRb1 in hippocampus tissue was detected by LC-MS/MS technique in figure S1. Table S2. The F value and P value in multiple comparisons of Fig. 1. Table S3. The F value and P value in multiple comparisons of Fig. 2. Table S4. The F value and P value in multiple comparisons of Fig. 3. Tablse S5. The F value and P value in multiple comparisons of Fig. 4. Table S6. The F value and P value in multiple comparisons of figure S2. Table S7. The F value and P value in multiple comparisons of Fig. S3Table S8. The F value and P value in multiple comparisons of Fig. 5. [file 12974_2021_2185_MOESM4_ESM.zip › 12974_2021_2185_MOESM4_ESM/Table S7.docx]

**Table 8．The F value and P value in multiple comparisons of figure 5**

**Fig. S3. The activation of PPARγ increase the DCX protein expression in GRb1-treated microglia *in vitro*.**

| *figure* | group | F or T | P | N |
| --- | --- | --- | --- | --- |
| *Figure B* | LPS vs. Ctrl |  | 0.9999 |  |
|  | GRb1 vs. Ctrl | 0.849 | 0.9416 | 5 |
|  | LPS+GRb1 vs. LPS |  | 0.4891 |  |
|  | LPS+GRb1+GW vs. LPS+GRb1 | 2.198 | 0.0591 | 5 |
| *Figure C* | LPS vs. Ctrl |  | 0.0328 |  |
|  | GRb1 vs. Ctrl | 0.328 | 0.6358 | 5 |
|  | LPS+GRb1 vs. LPS |  | 0.0030 |  |
|  | LPS+GRb1+GW vs. LPS+GRb1 | 2.944 | 0.0186 | 5 |
