# Supplementary material for: Ginsenoside Rb1 induces a pro-neurogenic microglial phenotype via PPARγ activation in male mice exposed to chronic mild stress
Source: J Neuroinflammation. 2021 Aug 9;18:171. doi: 10.1186/s12974-021-02185-0 (PMC8353817; doi:10.1186/s12974-021-02185-0)
Supplement: Supplementary file 4 — Additional file 4: Table S1. The concentration of GRb1 in hippocampus tissue was detected by LC-MS/MS technique in figure S1. Table S2. The F value and P value in multiple comparisons of Fig. 1. Table S3. The F value and P value in multiple comparisons of Fig. 2. Table S4. The F value and P value in multiple comparisons of Fig. 3. Tablse S5. The F value and P value in multiple comparisons of Fig. 4. Table S6. The F value and P value in multiple comparisons of figure S2. Table S7. The F value and P value in multiple comparisons of Fig. S3Table S8. The F value and P value in multiple comparisons of Fig. 5. [file 12974_2021_2185_MOESM4_ESM.zip › 12974_2021_2185_MOESM4_ESM/Table S8.docx]

**Table 5．The F value and P value in multiple comparisons of figure 5**

**Fig. 5. PPARγ activation mediates effects of GRb1-treated microglia *in vitro*.**

| *figure* | group | F or T | P | N |
| --- | --- | --- | --- | --- |
| *Figure 5B* | LPS vs. Ctrl |  | 0.0001 |  |
|  | GRb1 vs. Ctrl | 1.675 | 0.0882 | 6 |
|  | LPS+GRb1 vs. LPS |  | <0.0001 |  |
|  | LPS+GRb1+GW vs. LPS+GRb1 | 5.691 | 0.0002 | 6 |
| *Figure 5C* | LPS vs. Ctrl |  | 0.0028 |  |
|  | GRb1 vs. Ctrl | 0.472 | 0.1873 | 6 |
|  | LPS+GRb1 vs. LPS |  | 0.0280 |  |
|  | LPS+GRb1+GW vs. LPS+GRb1 | 4.375 | 0.0014 | 6 |
| *Figure 5E-1* | LPS vs. Ctrl |  | 0.0007 |  |
|  | GRb1 vs. Ctrl | 4.899 | 0.2305 | 5 |
|  | LPS+GRb1 vs. LPS |  | 0.0003 |  |
|  | LPS+GRb1+GW vs. LPS+GRb1 | 6.142 | 0.0003 | 5 |
| *Figure 5E-2* | LPS vs. Ctrl |  | 0.0002 |  |
|  | GRb1 vs. Ctrl | 1.296 | 0.5856 | 5 |
|  | LPS+GRb1 vs. LPS |  | <0.0001 |  |
|  | LPS+GRb1+GW vs. LPS+GRb1 | 7.819 | <0.0001 | 5 |
| *Figure 5F* | LPS vs. Ctrl |  | < 0.0001 |  |
|  | GRb1 vs. Ctrl | 16.850 | 0.9873 | 5 |
|  | LPS+GRb1 vs. LPS |  | < 0.0001 |  |
|  | LPS+GRb1+GW vs. LPS+GRb1 | 2.953 | 0.0112 | 5 |
| *Figure 5G* | LPS vs. Ctrl |  | < 0.0001 |  |
|  | GRb1 vs. Ctrl | 25.380 | 0.1650 | 5 |
|  | LPS+GRb1 vs. LPS |  | < 0.0001 |  |
|  | LPS+GRb1+GW vs. LPS+GRb1 | 2.607 | 0.0154 | 5 |
| *Figure 5J* | LPS vs. Ctrl |  | < 0.0001 |  |
|  | GRb1 vs. Ctrl | 20.280 | 0.2200 | 5 |
|  | LPS+GRb1 vs. LPS |  | < 0.0001 |  |
|  | LPS+GRb1+GW vs. LPS+GRb1 | 1.514 | 0.1523 | 5 |
| *Figure 5K* | LPS vs. Ctrl |  | < 0.0001 |  |
|  | GRb1 vs. Ctrl | 26.160 | 0.7954 | 5 |
|  | LPS+GRb1 vs. LPS |  | < 0.0001 |  |
|  | LPS+GRb1+GW vs. LPS+GRb1 | 5.004 | 0.0002 |  |
| *Figure 5L* | LPS vs. Ctrl |  | 0.7905 |  |
|  | GRb1 vs. Ctrl | 1.932 | 0.0076 | 5 |
|  | LPS+GRb1 vs. LPS |  | 0.1923 |  |
|  | LPS+GRb1+GW vs. LPS+GRb1 | 0.387 | 0.7039 | 5 |
| *Figure 5M* | LPS vs. Ctrl |  | 0.0001 |  |
|  | GRb1 vs. Ctrl | 29.610 | 0.4226 | 5 |
|  | LPS+GRb1 vs. LPS |  | < 0.0001 |  |
|  | LPS+GRb1+GW vs. LPS+GRb1 | 5.007 | 0.0001 | 5 |
| *Figure 5N* | LPS vs. Ctrl |  | 0.2553 |  |
|  | GRb1 vs. Ctrl | 1.193 | 0.9948 | 6 |
|  | LPS+GRb1 vs. LPS |  | 0.0732 |  |
|  | LPS+GRb1+GW vs. LPS+GRb1 | 0.123 | 0.9049 | 6 |
| *Figure 5O* | LPS vs. Ctrl |  | 0.0153 |  |
|  | GRb1 vs. Ctrl | 1.605 | 0.9997 | 6 |
|  | LPS+GRb1 vs. LPS |  | 0.0447 |  |
|  | LPS+GRb1+GW vs. LPS+GRb1 | 13.140 | < 0.0001 | 6 |
